# Machine Learning Differentiation of Autism Spectrum Sub-Classifications

**DOI:** 10.1007/s10803-023-06121-4

**Published:** 2023-09-26

**Authors:** R Thapa, A Garikipati, M Ciobanu, NP Singh, E Browning, J DeCurzio, G Barnes, FA Dinenno, Q Mao, R Das

**Affiliations:** Montera, Inc dba Forta, 548 Market St, PMB 89605, San Francisco, CA USA

**Keywords:** Machine learning, Autism, Diagnostics, Classification

## Abstract

**Purpose:**

Disorders on the autism spectrum have characteristics that can manifest as difficulties with communication, executive functioning, daily living, and more. These challenges can be mitigated with early identification. However, diagnostic criteria has changed from DSM-IV to DSM-5, which can make diagnosing a disorder on the autism spectrum complex. We evaluated machine learning to classify individuals as having one of three disorders of the autism spectrum under DSM-IV, or as non-spectrum.

**Methods:**

We employed machine learning to analyze retrospective data from 38,560 individuals. Inputs encompassed clinical, demographic, and assessment data.

**Results:**

The algorithm achieved AUROCs ranging from 0.863 to 0.980. The model correctly classified 80.5% individuals; 12.6% of individuals from this dataset were misclassified with another disorder on the autism spectrum.

**Conclusion:**

Machine learning can classify individuals as having a disorder on the autism spectrum or as non-spectrum using minimal data inputs.

**Supplementary Information:**

The online version contains supplementary material available at 10.1007/s10803-023-06121-4.

Diagnostic criteria and categorization for disorders on the autism spectrum within the Diagnostic and Statistical Manual of Mental Disorders, 4th Edition (DSM-IV) were changed in the 2013 Diagnostic and Statistical Manual of Mental Disorders, 5th Edition (DSM-5). At this point, the term autism spectrum disorder (ASD) was introduced to replace older criteria and categorization, and to become a standalone diagnosis. Hereafter, we refer to the autism spectrum disorder as “ASD” in the context of the DSM-5 diagnostic criteria or to discuss ASD generally, outside of the context of DSM-IV diagnostic criteria. However, and owing to significantly greater availability, DSM-IV-based data and categorization are used for the purposes of analysis in this paper. Further, for the purposes of this paper, individuals who have not been identified as having a disorder on the autism spectrum are referred to as “non-spectrum.”

ASD is neurodevelopmental in origin and has multifaceted and diverse manifestations that impact multiple domains of an individual’s life (Abbas et al., 2020; Masi et al., 2017). While there are no known causes that influence the likelihood of being diagnosed with ASD, some research has suggested that genetics and exposure to certain environmental conditions may be correlated with a diagnosis of ASD (Gaugler et al., 2014). In addition to impacting the individual having the disorder, families and caregivers frequently experience economic and psychological repercussions (Buescher et al., 2014; Fewster et al., 2019). Though most of the current epidemiological evidence regarding direct and indirect costs associated with ASD is historic due to lack of longitudinal estimates of costs associated with DSM-5-based diagnoses, a study by Leigh et al. estimates that the projected economic costs associated with ASD by 2025 in the United States (US) to be $461B, which encompasses healthcare expenditures, expenditures not related to medical care (e.g., education services), and employment loss for individuals with the disorder and their caregivers (Leigh & Du, 2015). Significantly higher and continually increasing prevalence of ASD among individuals in general, including individuals with diverse ethnic makeup and females in comparison to epidemiological estimates from the previous 20 years (Maenner, 2023), may lead to more healthcare and service utilization. These increases have the potential to drive up costs associated with ASD (Matin et al., 2022). However, accrual and analysis of more robust longitudinal data will be needed to establish the true financial burden of ASD within the DSM-5 diagnostic era. Individuals with ASD also experience an increased number of health risks and challenges, including comorbidities and excess mortality at a rate of 2–10 times more than individuals without ASD, owing to comorbid conditions, suicide, and accidental injury (Guan & Li, 2017; Hirvikoski et al., 2016). As many of these comorbidities and social risk-taking behaviors increased in prevalence after diagnostic criteria was updated within DSM-5 (e.g., attention deficit hyperactivity disorder (ADHD) and risky driving, respectively) (Romero et al., 2016), it is possible that, as more longitudinal data becomes available, this excess mortality may change. For example, DSM-5 allows ADHD diagnosis as a comorbidity for individuals with ASD. Such a diagnosis may provide the opportunity of psychopharmacological treatment of symptoms of overactivity and inattention, which has been shown to reduce traits of ADHD (inattentiveness, impulsive behaviors) and improve co-occuring conditions (substance use disorders, mental health disorders, etc.). In turn, this may improve life expectancy (Barkley & Fischer, 2019; Boland et al., 2020). However, absent longitudinal mortality data, excess mortality for individuals with ASD by the DSM-5 criteria is speculative.

As part of the diagnostic process, screening questionnaires such as the Autism Diagnostic Interview-Revised (ADI-R) and the Social Communication Questionnaire (SCQ) are used to identify characteristics of ASD (Eaves et al., 2006; Papanikolaou et al., 2009). However, they can not be used as standalone tools to yield a diagnosis as they require additional clinician assessment and interpretation (Eaves et al., 2006; Papanikolaou et al., 2009). Further, the combination of these assessments with additional clinical analysis is a time consuming process. Disagreement within the scientific communities regarding DSM-IV criteria versus DSM-5 criteria for diagnosis, classification, and treatment recommendations for ASD adds an additional layer of complexity to the diagnostic process, though both have pros and cons in terms of their diagnostic capabilities.

DSM-IV encompassed distinct disorders on the autism spectrum, including autistic disorder, Asperger’s disorder^1^, and pervasive developmental disorder - not otherwise specified (PDD-NOS) (Neal et al., 2012; Singer, 2012; Volkmar & Reichow, 2013), allowing for a broad range of presentations and levels of severity. DSM-5 eliminated these DSM-IV classifications and established a new diagnostic category termed ASD. Under DSM-5 diagnostic criteria, individuals with ADHD (previously precluded from an ASD diagnosis) and individuals with the DSM-IV categorization of Asperger’s disorder were absorbed into the ASD diagnosis. This, and the increased identification of ASD among females and individuals of non-white ethnic backgrounds that occurred in more recent years (Maenner, 2023) may have contributed to broadening the overall size of the population of individuals with ASD. Concurrent with increasing ASD prevalence under DSM-5, the overall patient outcomes have changed as well. Particularly for individuals with co-occuring ASD and ADHD, DSM-5 provided the opportunity of therapeutic treatments (pharmacological and psychological) to address symptoms that overlap between ADHD and ASD, which may have led to improved patient outcomes (Antshel & Russo, 2019). Additionally, individuals that would have been diagnosed with ASD later under DSM-IV, due to atypical presentation of characteristics (e.g., those with Asperger’s disorder) (Mandell et al., 2005), could experience improved outcomes if, as the increased prevalence of ASD suggests, these individuals can be captured with the DSM-5 criteria earlier.

The DSM-5 updated diagnostic criteria was intended to reflect the distinct and consistent presentation of two symptoms of disorders on the autism spectrum, social/communication and restrictive and repetitive behaviors (RRBs) and is viewed by some members of the medical community as being a more streamlined and less complex approach to diagnose ASD (Mahjouri & Lord, 2012). One con, however, as other members of the medical community point out, is that the ASD diagnostic criteria within DSM-5 were more restrictive than DSM-IV criteria (Hosseini & Molla, 2023; Singer, 2012). This resulted in many individuals being unable to meet the clinical criteria for an ASD diagnosis (Kulage et al., 2014; McPartland et al., 2012; Neal et al., 2012; Singer, 2012; Volkmar & McPartland, 2014). Without such a diagnosis, individuals may experience limitations on access to healthcare services and educational disability services, which, in turn, can result in poor social and/or academic outcomes, lower rates of employment, and decreased assistance in eventual independent living (Lobar, 2016).

DSM-IV, in contrast to DSM-5, provided over 2,000 combinations of criteria to diagnose the three disorders (i.e., autistic disorder, Asperger’s disorder, PDD-NOS) that are now encompassed under the single diagnostic category of ASD in DSM-5 (Lenart & Pasternak, 2021; Volkmar & Reichow, 2013). This provided flexibility in making a diagnosis to account for atypical presentations, including age at symptom onset, as well as language and intellectual abilities (Mazurek et al., 2017). Under DSM-5, there are 11 criteria combinations, which led to many individuals not meeting the new threshold for a diagnosis of ASD (Lenart & Pasternak, 2021; Volkmar et al., 2021; Volkmar & Reichow, 2013). The sensitivity for identifying several disorders on the autism spectrum, including individuals who would have been diagnosed with PDD-NOS and Asperger’s disorder under DSM-IV were generally low (0.28 and 0.25, respectively) (McPartland et al., 2012). Additionally, subsequent to implementation of DSM-5, the stability of PDD-NOS and Asperger’s disorder diagnoses decreased (Bent et al., 2017). Research has also demonstrated that females, young children, non-cognitively impaired individuals, and individuals over 8 years of age are disproportionately unable to meet DSM-5 criteria, even if they have a DSM-IV diagnosis (Mazurek et al., 2017; Neal et al., 2012; Singer, 2012). In response to expert criticism on the impact of these new criteria, particularly as it relates to access to health services, multiple steps were taken. First, DSM-5 “grandfathered in” patients with a previous diagnosis of autistic disorder, Asperger’s disorder, or PDD-NOS under DSM-IV (Kulage et al., 2014; McPartland et al., 2012; Neal et al., 2012; Singer, 2012; Volkmar & McPartland, 2014; Volkmar & Reichow, 2013). This provided a resolution for patients with an existing DSM-IV diagnosis but did not address the issue of individuals who had yet to receive a DSM-IV diagnosis and were unable to meet DSM-5 criteria (Volkmar et al., 2021). Second, the National Institute of Mental Health developed the Research Domain Criteria (RDoC) framework (Mandy, 2018). RDoC encourages the evaluation of individuals for mental health conditions with consideration given to the numerous variables that may contribute to a disorder (*About RDoC*, n.d.; Garvey et al., 2016; Mandy, 2018). These include symptoms or behaviors that could potentially be attributed to multiple, and potentially inter-related disorders; the heterogenous range of functioning that individuals display within different domains of functioning; and potential causation within different types of environments (*About RDoC*, n.d.; Garvey et al., 2016). However, the RDoC framework is not intended to contribute to the diagnostic process. Rather, it is designed for use in and to inform research, such that it encourages exploratory research as opposed to research that is restricted by traditional diagnostic classifications (Garvey et al., 2016; Knott et al., 2021).

There is ample literature focused on the challenges associated with the redefined ASD diagnostic criteria under DSM-5. Mazurek et al. conducted a clinical study of pediatric patients with features of ASD to investigate agreement between DSM-IV and DSM-5 criteria (Mazurek et al., 2017). Out of 439 patients who were being assessed at multiple autism-focused centers, 278 participants were eligible for a DSM-IV diagnosis of a disorder on the autism spectrum and 249 were eligible for a DSM-5 diagnosis of ASD. Only 1 participant with a DSM-5 diagnosis of ASD did not meet the DSM-IV diagnostic criteria, whereas 30 individuals with a DSM-IV diagnosis did not meet the DSM-5 criteria for a diagnosis of ASD (Mazurek et al., 2017). Further, 20% and 75% of individuals who were diagnosed with Asperger’s disorder or PDD-NOS, respectively, did not meet the diagnostic criteria for a DSM-5 diagnosis of ASD (Mazurek et al., 2017).

Machine learning (ML) is a powerful tool in personalized medicine and has been validated for a variety of prediction and diagnostic tasks for acute and chronic medical issues (*Cognoa - Leading the Way for Pediatric Behavioral Health*, n.d.; Hassan et al., 2022; Lam et al., 2022; Thapa et al., 2022; Tso et al., 2022; Varma et al., n.d.). Recent research has explored the use of ML to facilitate earlier diagnosis of individuals with ASD by addressing logistical and practical diagnostic barriers, to identify a minimal feature set of inputs that achieve high accuracy in aiding ASD diagnosis to determine if a simplified diagnosis process is feasible, and to design ASD screening tools (Abbas et al., 2020; *Cognoa - Leading the Way for Pediatric Behavioral Health*, n.d.; Duda et al., 2016; Kosmicki et al., 2015; Megerian et al., 2022; Tariq et al., 2018; Wall et al., 2012).

In the present study, we examine the use of ML to classify individuals as either having autistic disorder, Asperger’s disorder, PDD-NOS (based on DSM-IV criteria alone) or as not having such a disorder (i.e., non-spectrum) by using readily available patient information and with minimal demands on clinical teams and clinical workflow. Figure 1 depicts the workflow of the prediction algorithm in clinical practice. The use of DSM-IV criteria was not intended to serve as a challenge or criticism to the validity of DSM-5. Rather, owing to limited access to DSM-5 ASD diagnostic data, only DSM-IV-based data were used in this study. Our proof-of-concept study provides the basis for future work using ML-based tools to evaluate DSM-5 diagnoses. Further, our study may provide an avenue for the identification of individuals with one of these three disorders (i.e., autistic disorder, Asperger’s disorder, PDD-NOS) that may not be classified as having ASD using DSM-5 criteria. This could facilitate an additional in-depth assessment and earlier interventions, which are vital for improving outcomes for individuals with ASD or previously associated disorders of the autism spectrum. On a macro-level, our research on the use of ML to identify patients that fall within a DSM-IV-based ASD classification may contribute to a body of research regarding comparative effectiveness approaches for patient evaluation (Esmail et al., 2020), as our machine learning algorithm (MLA) can be broadly implemented into electronic health record (EHR) systems, runs autonomously, and may provide a cost-effective and personalized approach for evaluation of patients with symptoms of neurodevelopmental disorders.

**Fig. 1:**
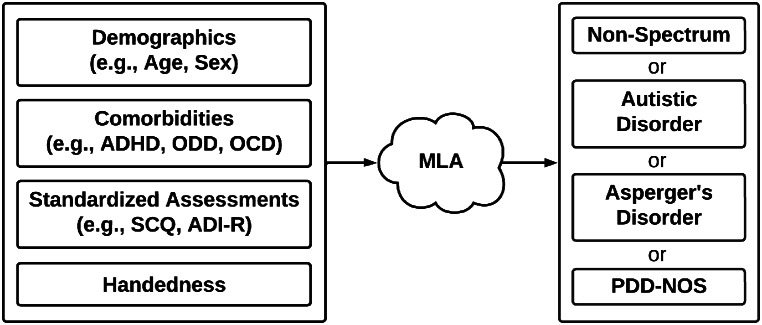
Workflow of the prediction algorithm in clinical practice. Inputs (depicted on the left) for an individual are analyzed by the MLA to create a prediction about the classification in which that particular individual will fall. The four possible classifications (i.e., MLA outputs) are depicted on the right. Abbreviations: attention deficit hyperactivity disorder (ADHD), oppositional defiant disorder (ODD), obsessive-compulsive disorder (OCD), Social Communication Questionnaire (SCQ), Autism Diagnostic Interview-Revised (ADI-R), machine learning algorithm (MLA), pervasive developmental disorder - not otherwise specified (PDD-NOS). Figure created using Lucidchart

## Methods

**Fig. 2:**
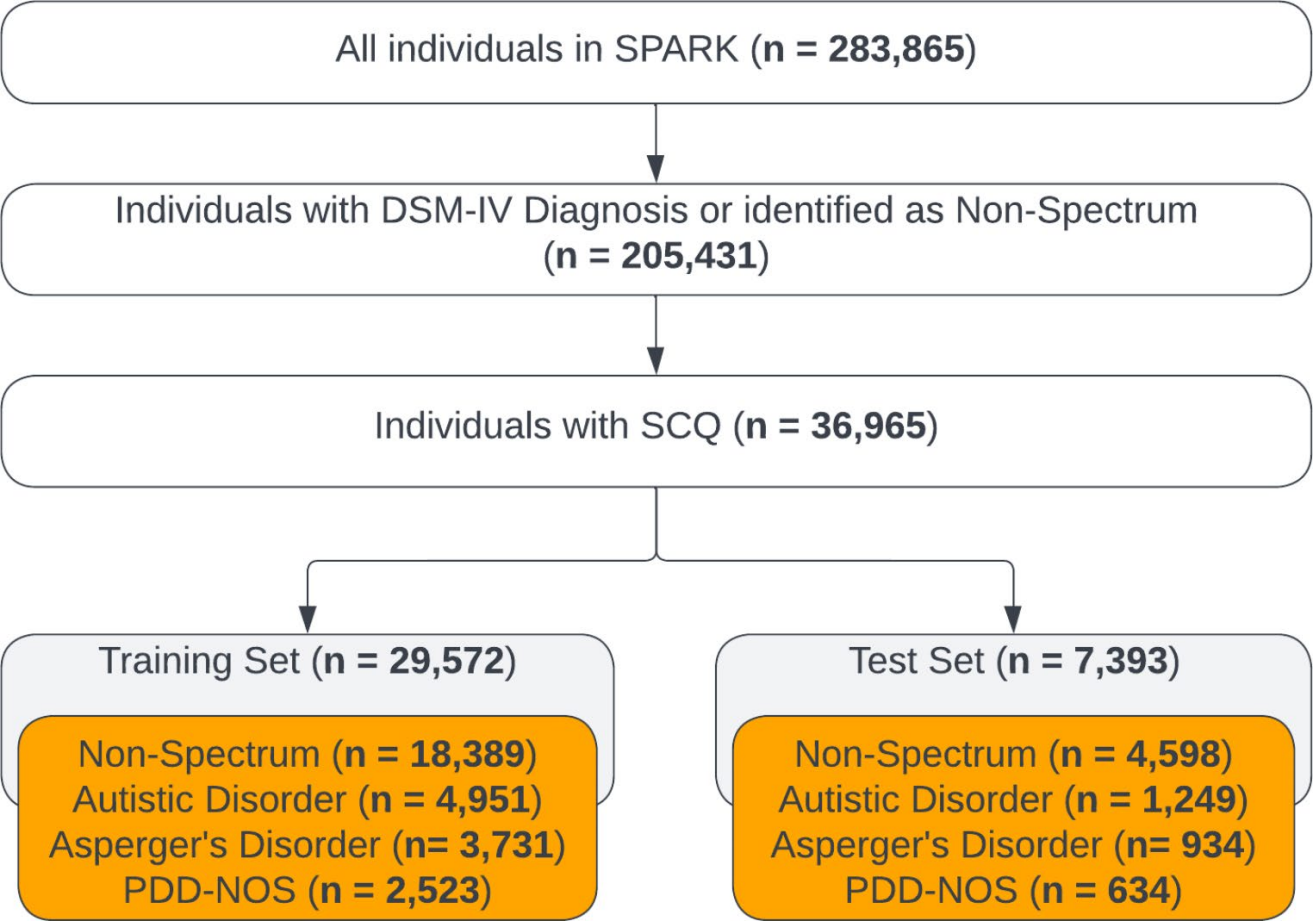
Attrition chart for primary dataset. To build our primary dataset, we selected individuals with a DSM-IV diagnosis of a disorder on the autism spectrum and those identified as non-spectrum. The individuals with a DSM-IV diagnosis were also required to have an SCQ score. The resulting primary dataset was split 80/20 into training and testing datasets, respectively. Abbreviations: Diagnostic and Statistical Manual of Mental Disorders, 4th Edition (DSM-IV), Social Communication Questionnaire (SCQ), pervasive developmental disorder - not otherwise specified (PDD-NOS). Figure created using Lucidchart

Data for this study were obtained from two publicly available datasets: Simons Foundation Powering Autism Research for Knowledge (SPARK) and Autism Brain Imaging Data Exchange (ABIDE). SPARK, the primary dataset used for development and validation of our MLA, contains phenotypic data from over 280,000 individuals and contains demographic information, comorbidities, and diagnostic assessment tests (*SPARK: A US Cohort of 50,000 Families to Accelerate Autism Research - PMC*, n.d.) ABIDE, the supplementary dataset used to further support our main results, is primarily a database containing functional magnetic resonance imaging data, but also includes some types of phenotypic data (e.g., demographic information, comorbidities, medication status, and intelligence quotient (IQ) assessment with a breakdown of full scale intelligence quotient (FIQ), verbal intelligence quotient (VIQ), and performance intelligence quotient (PIQ). (Martino et al., 2014). In order to be identified as having autistic disorder, Asperger’s disorder, or PDD-NOS, both datasets required individuals to have a professional diagnosis of a condition based on DSM-IV criteria. (Martino et al., 2014; SPARK: A US Cohort of 50,000 Families to Accelerate Autism Research - PMC, n.d.). These diagnoses, or being identified as non-spectrum by a lack of such diagnostic codes within the datasets, were utilized as the ground truth for our models. Both datasets underwent an initial filtration, where only the individuals who were either identified as non-spectrum or had a DSM-IV diagnosis were selected. Similarly, individuals having a DSM-IV diagnosis but who did not have relevant diagnostic assessments data, such as SCQ (SPARK) or ADI-R (ABIDE), were also removed. The final dataset included 36,965 individuals for SPARK and 1,595 individuals for ABIDE (Fig. 2 and Online Resource Supplementary Fig. 1, respectively).

Individuals identified as non-spectrum held the highest prevalence in both datasets, with ~ 62% in SPARK (primary dataset) and ~ 55% in ABIDE (supplementary dataset), whereas PDD-NOS held the lowest prevalence in both datasets, with ~ 8% in SPARK and ~ 6% in ABIDE. SPARK includes data from a pediatric population with age range 2–19, with mean age of 9.2 and median age of 9.0 [Online Resource Supplementary Fig. 2], whereas ABIDE includes data from a general population with age range of 5–64, with mean age of 15.6 and median age of 13.2 [Online Resource Supplementary Fig. 3].

For the SPARK dataset, a required institutional review board (IRB) determined the project to be exempt from the US federal regulations for the protection of human subjects (22-MONT-101). For the ABIDE dataset, no IRB review or approval was required as follows. Within ABIDE, as data were de-identified to maintain compliance with Health Insurance Portability and Accountability Act (HIPAA), this did not constitute human subjects research per 45 US Code of Federal Regulations 46.102.

The filtered dataset was randomly split into a training dataset and a hold-out testing dataset (i.e., testing dataset), such that 80% of the data was in the training dataset and the remaining 20% of the data was in the testing dataset. The training and testing datasets remained completely independent of each other. In other words, there was no overlap between the individuals in the training dataset and the individuals in the testing dataset. The training dataset was used to train and optimize the MLA and the testing dataset was used solely to evaluate model results to determine MLA’s efficacy.

Two types of models were developed to highlight the ability of the ML models to achieve different tasks: a binary classifier and a multi-class classifier. The binary classifier showcases the ability of the ML model to discriminate between individuals with any DSM-IV disorder on the autism spectrum vs. individuals who are non-spectrum (e.g., autistic disorder vs. non-spectrum, Asperger’s disorder vs. non-spectrum, PDD-NOS vs. non-spectrum), thus enabling providers to administer further assessment and develop an appropriate therapy plan. The multi-class classifier provides additional insight on the specific DSM-IV disorder on the autism spectrum a patient may have, which can further inform the therapy plan and allow it to be tailored to the patient’s specific needs. The specific utility of each of the binary classifier and multi-class classifier showcases a potential workflow of using the models in practice in a real-world scenario.

During the training process, a gradient-boosted tree algorithm was utilized for the multi-class classification of individuals having autistic disorder, Asperger’s disorder, PDD-NOS, or non-spectrum individuals. Similarly, a gradient-boosted tree algorithm was also utilized to build the binary classification model which classified individuals as having any DSM-IV disorder on the autism spectrum. The binary and multi-class models are both classifier models, therefore enabling the use of similar modeling techniques and the same training and testing sets to train the models. Recent studies have shown that gradient-boosted tree algorithms can be used for a variety of clinical prediction tasks, including sepsis onset prediction, long-term care fall prediction, non-alcoholic steatohepatitis or fibrosis, neurological decompensation, classification of appropriate treatment plan intensity for ASD patients, and have demonstrated strong algorithm performance (Barton et al., 2019; Ghandian et al., 2022; S.-H. Kim et al., 2021; Li et al., 2021; Maharjan et al., 2023; Mao et al., 2018; Thapa et al., 2022). Tree-based models utilize the values of a subset of inputs to build a path to a specific classification (e.g., autistic disorder classification, Asperger’s disorder classification, PDD-NOS classification, non-spectrum classification) in which a particular set of inputs belongs. This set of paths connecting the values of the subset of features to a particular classification is known as a decision tree. The process of constructing trees is repeated to develop a series of decision trees which are utilized in combination to determine the final output of the model. As the complete model contains several trees, each with a subset of the input data and input features, it is able to perform classifications that account for the heterogeneity of feature values that an individual diagnosed with a DSM-IV disorder on the autism spectrum may present. As an example, while one individual diagnosed with a DSM-IV disorder on the autism spectrum may present with relatively more verbal communication deficits, another individual with an identical diagnosis may present with relatively more RRBs. The combination of multiple trees within the model can accurately discriminate between the different presentations and diagnose both individuals with the correct classification per the DSM-IV criteria.

A hyperparameter optimization was performed on the training dataset using a 5-fold cross-validation grid search and confirmed by evaluating the area under the receiver operating characteristic curve (AUROC) for different combinations of hyperparameters included in the grid search. The hyperparameters which were optimized included maximum tree depth, number of estimators, L1 regularization, and learning rate. The optimal hyperparameters were identified as the hyperparameters which, in combination, resulted in the strongest performing model across the cross-validations folds [Online Resource Supplementary Table 1].

Following model training, performance was evaluated on the 20% hold-out testing dataset (i.e., testing dataset). Primary performance metrics included AUROC, sensitivity, specificity, positive predictive value (PPV), and negative predictive value (NPV), which were all evaluated at a single operating sensitivity point, set at 0.85. The single operating sensitivity point was selected such that all classifications were evaluated at a similar sensitivity, allowing for a more direct comparison between the performance of the model for each classification. In addition, 95% confidence intervals (CIs) were computed for each metric with the method described in the Statistics section below. A SHapely Additive exPlanations (SHAP) analysis (Lundberg & Lee, 2017) was performed to evaluate the importance of each feature for generating model output by examining the ways in which the feature values of each element of the training dataset affect the classification of the training examples. The SHAP plot ranks features by importance to model predictions top to bottom in the decreasing order of importance.

The final set of input features that were used to train and test the MLA are shown in Table 1. The inputs for the supplementary dataset can be found in the Supplement [Online Resource Supplementary Table 2]. Though these inputs shared many features across both datasets, the primary dataset used SCQ as the scoring system, whereas the supplementary dataset used ADI-R. Both datasets contained five comorbidities, all of which were identical - with the exception of language disorder (primary dataset) and phobias (supplementary dataset). Further, only the supplementary dataset contained information about current medication usage and IQs. It should be noted that DSM-IV did not allow for a concurrent diagnosis of ADHD (one of the included comorbidities) (Ghanizadeh, 2010). Thus, it is likely that the presence of ADHD diagnoses in the datasets was the result of ADHD diagnoses delivered starting in 2013 (when DSM-5 criteria were instated) to patients already holding a diagnosis under DSM-IV of autistic disorder, Asperger’s disorder, or PDD-NOS. Additionally, although perhaps less likely, it may be possible that some individuals were diagnosed after 2013 under DSM-IV with autistic disorder, Asperger’s disorder, or PDD-NOS, and also received a diagnosis of ADHD.

**Table 1:** Data used from the primary dataset to generate algorithm inputs. Inputs included demographic information, assessment data, comorbidities, and handedness. Abbreviations: attention deficit hyperactivity disorder (ADHD), machine learning algorithm (MLA), oppositional defiant disorder (ODD), Social Communication Questionnaire (SCQ).

MLA Inputs	
Demographics ● Age ● Sex	Comorbidities Attention Deficit Hyperactivity Disorder (ADHD) Oppositional Defiant Disorder (ODD) Obsessive-Compulsive Disorder (OCD) Anxiety/Generalized Anxiety Disorder Language Disorder
Assessment Data ● Social Communication Questionnaire (SCQ) ○ 40 Questions ○ Social Total ○ Restricted, Repetitive Behaviors Total ○ Communication Total ○ Total	Handedness ● Left ● Right ● Ambidextrous

### Statistics

To calculate the CI for the AUROC, a bootstrapping method was utilized, in which a random sample of patients was selected from the testing dataset and the AUROC was computed solely with those patients (Liu et al., 2022, 2016; *On Bootstrapping the ROC Curve | Proceedings of the 21st International Conference on Neural Information Processing Systems*, n.d.). This process of randomly selecting patients and computing an AUROC was repeated 1,000 times. Random sampling of testing dataset patients was performed with replacement. From these 1,000 bootstrapped AUROC values, the middle 95% range was selected to be the 95% CI for the AUROC. As the sample size of each of our datasets (i.e., primary dataset and supplementary dataset) was sufficiently large, the CIs for other metrics were calculated using normal approximation (Hazra, 2017). Similarly, the difference between various groups was studied using a two-sided t-test with a 95% significance level.

## Results

**Fig. 3:**
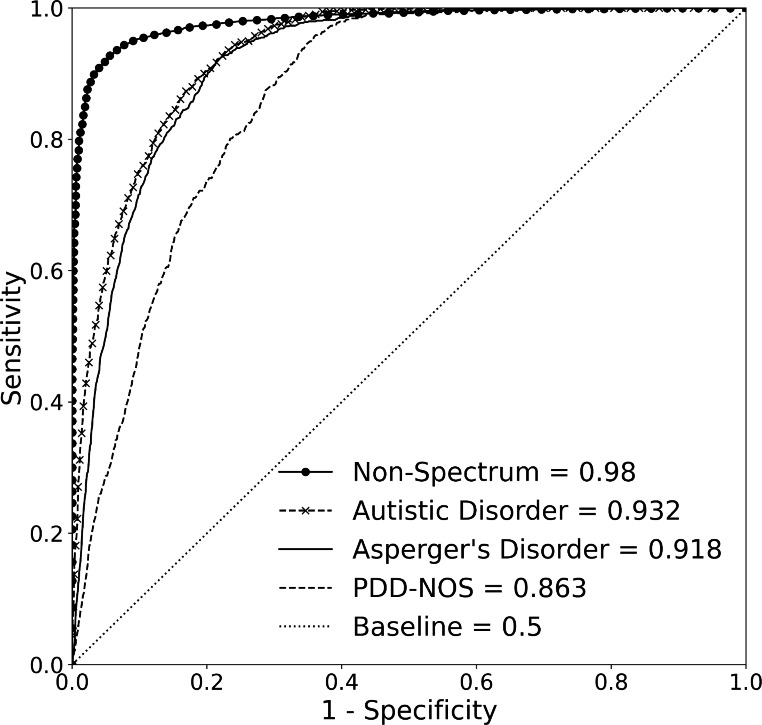
Area under receiver operating characteristic curve (AUROC) demonstrating the machine learning algorithm’s (MLA’s) performance for classifying individuals into the four output classifications using Simons Foundation Powering Autism Research for Knowledge (SPARK) testing dataset. For all four classifications, the MLA performed better than the baseline. The baseline curve represents a model that is not able to differentiate between classifications, effectively equivalent to random coin-flip. Abbreviation: pervasive developmental disorder - not otherwise specified (PDD-NOS). Figure created using Seaborn and Matplotlib in Python

Demographic information and the total number of individuals in each classification in the primary training and testing datasets are shown in Table 2 and Online Resource Supplementary Tables 3, respectively. Online Resource Supplementary Tables 4 and 5 contain the corresponding information for the supplementary training and testing datasets, respectively. Table 3; Fig. 3 present results from the primary testing dataset. Online Resource Supplementary Fig. 4 and Online Resource Supplementary Table 6 contain the corresponding information for the supplementary testing dataset^2^.

**Table 2:** Demographics table using Simons Foundation Powering Autism Research for Knowledge (SPARK) training dataset showing the breakdown of individuals in each classification by age, gender, and race/ethnicity. This table also shows the comorbidities present within each classification. Abbreviations: attention deficit hyperactivity disorder (ADHD), obsessive-compulsive disorder (OCD), oppositional defiant disorder (ODD), pervasive developmental disorder - not otherwise specified (PDD-NOS).

Category	Demographics	TRAINING DATASET (N = 29,572)			
		Non-Spectrum (N = 18,389)	Autistic Disorder (N = 4,951)	Asperger’s Disorder (N = 3,731)	PDD-NOS (N = 2,523)
**Age (years)**	2–4	2906 (15.8%)	658 (13.3%)	58 (1.5%)	108 (4.3%)
	4–13	11260 (61.2%)	3116 (62.9%)	2002 (53.7%)	1444 (57.2%)
	13–20	4223 (23.0%)	1177 (23.8%)	1671 (44.8%)	971 (38.5%)
**Gender**	Male	8980 (48.8%)	3915 (79.1%)	2914 (78.1%)	1931 (76.5%)
	Female	9409 (51.2%)	1036 (20.9%)	817 (21.9%)	592 (23.5%)
**Race/** **Ethnicity**	White and Non-Hispanic	4672 (25.4%)	2266 (60.7%)	2485 (50.2%)	1379 (54.7%)
	Black and Non-Hispanic	496 (2.7%)	148 (4.0%)	395 (8.0%)	148 (5.9%)
	Asian and Non-Hispanic	315 (1.7%)	67 (1.8%)	140 (2.8%)	70 (2.8%)
	Hispanic	1254 (6.8%)	428 (11.5%)	861 (17.4%)	344 (13.6%)
	Native American	131 (0.7%)	110 (2.9%)	115 (2.3%)	48 (1.9%)
	Native Hawaiian	45 (0.2%)	25 (0.7%)	31 (0.6%)	13 (0.5%)
	Others	68 (0.4%)	18 (0.5%)	45 (0.9%)	19 (0.8%)
	Unknown	11408 (62.0%)	669 (17.9%)	879 (17.8%)	502 (19.9%)
**Comorbidities**	ADHD	2856 (15.5%)	1595 (32.2%)	2129 (57.1%)	1168 (46.3%)
	ODD	486 (2.6%)	329 (6.6%)	533 (14.3%)	279 (11.1%)
	OCD	314 (1.7%)	438 (8.8%)	519 (13.9%)	255 (10.1%)
	Anxiety	1651 (9.0%)	823 (16.6%)	1314 (35.2%)	622 (24.7%)
	Language Disorder	1482 (8.1%)	3213 (64.9%)	804 (21.5%)	1398 (55.4%)

**Table 3:** Area under the receiver operating characteristic curve (AUROC) demonstrating classification performance of the machine learning algorithm in each of the four classifications in the primary testing dataset as determined by the AUROC, sensitivity, specificity, positive predictive value, negative predictive value. All metrics include a 95% confidence interval. The prevalence of each classification using the primary testing dataset is also shown. Abbreviations: confidence interval (CI), negative predictive value (NPV), positive predictive value (PPV), pervasive developmental disorder - not otherwise specified (PDD-NOS).

	Non-Spectrum	Autistic Disorder	Asperger’s Disorder	PDD-NOS
**AUROC (95% CI)**	0.980 (0.978–0.983)	0.932 (0.926–0.938)	0.918 (0.911–0.926)	0.863 (0.853–0.874)
**Sensitivity (95% CI)**	0.858 (0.848–0.868)	0.850 (0.830–0.870)	0.851 (0.828–0.874)	0.852 (0.824–0.880)
**Specificity (95% CI)**	0.981 (0.976–0.986)	0.846 (0.837–0.855)	0.826 (0.817–0.836)	0.721 (0.710–0.732)
**PPV (95% CI)**	0.987 (0.983–0.990)	0.525 (0.503–0.547)	0.414 (0.392–0.436)	0.221 (0.205–0.238)
**NPV (95% CI)**	0.807 (0.794–0.821)	0.966 (0.961–0.971)	0.975 (0.97–0.979)	0.981 (0.978–0.985)
**Prevalence**	0.622	0.167	0.126	0.085

In the primary testing dataset, of the DSM-IV disorders on the autism spectrum, autistic disorder was most frequently observed in individuals who were aged 4–13, and whose race-ethnicity was White non-Hispanic, Native American, Native Hawaiian, and others (i.e., a race or ethnicity not classified as one of the categories in our demographics Table 2 and Online Resource Supplementary Table 3). A majority of individuals with a DSM-IV diagnosis of a disorder on the autism spectrum were male (> 75%), whereas females were more frequently classified as non-spectrum than males (51.7% and 48.3%, respectively), a difference that was statistically significant (p-value = 0.0007). Five comorbidities were observed in the primary dataset: ADHD, oppositional defiant disorder (ODD), obsessive compulsive disorder (OCD), anxiety, and language disorder. In the primary testing dataset (Online Resource Supplementary Table 3), ADHD was the most frequent comorbidity for individuals classified as non-spectrum (15.2%) and individuals with Asperger’s disorder (55.7%). For individuals classified as having autistic disorder or PDD-NOS, language disorder was the most frequent comorbidity and was present in 64.5% and 52.2% of individuals, respectively. The demographic distribution for the training dataset is shown in Table 2. As we have added in the footnotes^2^, within the demographics tables (Table 2 and Online Resource Supplementary Tables 3–5), the values for age are intended to be viewed across the classifications as opposed to across the demographic group. In the primary testing dataset, individuals having autistic disorder (n = 1249) had the highest prevalence in the age group of 4–13 years old (63.8%) followed by the prevalence in the age group of 13–20 years old (22.9%). Individuals from the primary testing dataset having Asperger’s disorder (n = 934) and PDD-NOS (n = 634), had the highest prevalence in the age group of 4–13 years old (54.6% and 57.3%, respectively). In the supplementary testing dataset, individuals having autistic disorder (n = 89) had the highest prevalence in the age group of 5–13 years old (39.3%). Individuals from the supplementary testing dataset having Asperger’s disorder (n = 34) and PDD-NOS (n = 20) had the highest prevalence in the age group of 5–13 years old (61.8% and 85%, respectively).

The MLA achieved strong performance for classifying individuals as non-spectrum in both datasets, with an AUROC of 0.980 in the primary testing dataset (CI: 0.978–0.983) (Fig. 3; Table 3) and 0.969 (CI: 0.951–0.984) in the supplementary testing dataset [Online Resource Supplementary Fig. 4 and Online Resource Supplementary Table 6]. In the primary testing dataset, this was followed by classification of autistic disorder, which achieved an AUROC of 0.932 (CI: 0.926–0.938). For classification of Asperger’s disorder and PDD-NOS, the MLA achieved AUROC values of 0.918 (CI: 0.911–0.926) and 0.863 (CI: 0.853–0.874), respectively. All algorithms substantially outperformed the baseline (AUROC of 0.500). Our results displayed in Table 3 as well as in Online Resource Supplementary Table 6 present the performance of the MLA when the model is tuned to a sensitivity of 0.85 in order to have a consistent set of comparisons across the different classifications. A fixed sensitivity was selected to prioritize true positive classifications and limit false negative classifications. The aim of this fixed sensitivity was to prevent false negatives as much as possible. While a false positive result may prompt further investigation, a false negative classification may lead to a lack of further analysis, which could impactfully diminish the chances of diagnosing that particular individual.

**Fig. 4:**
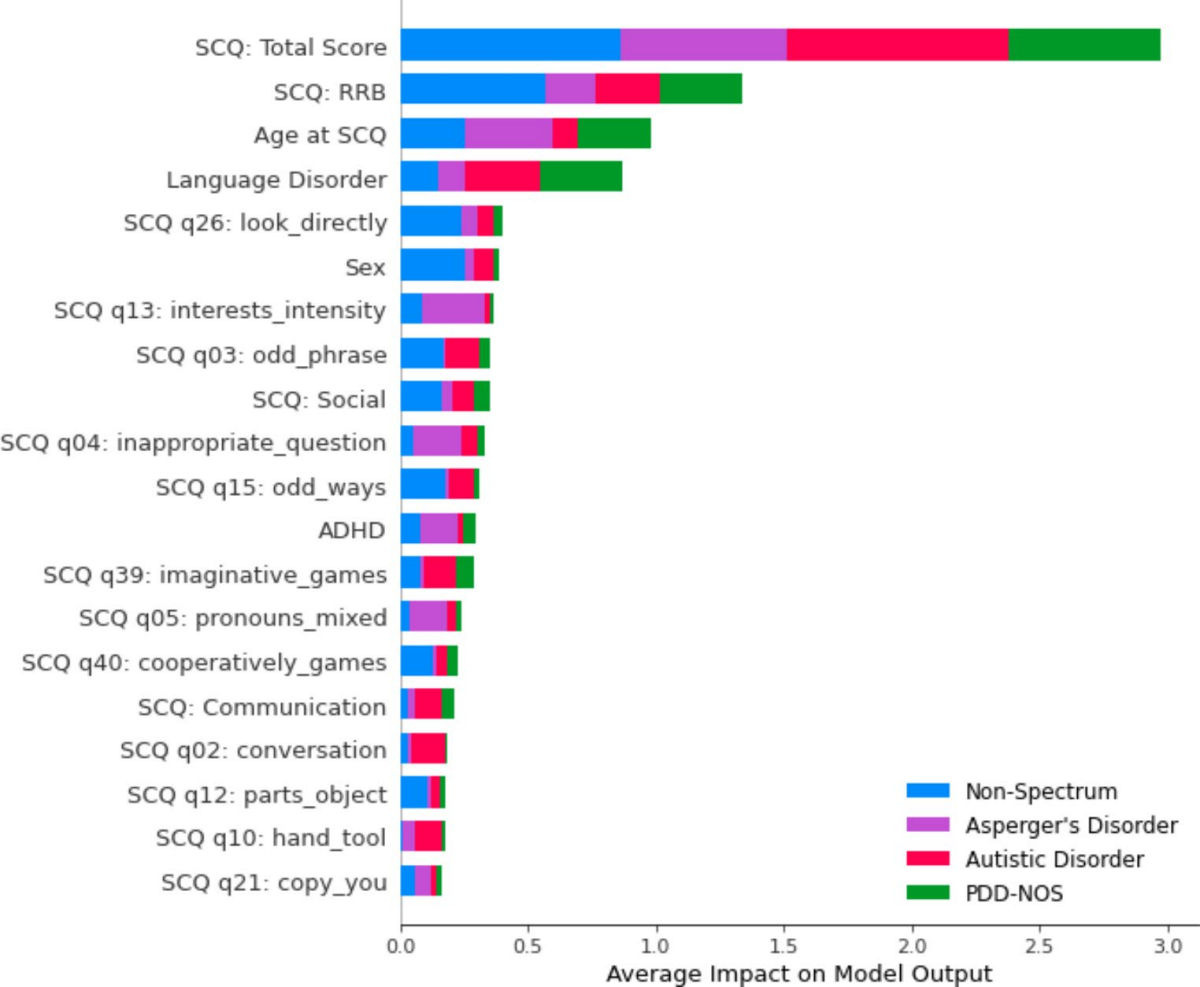
Feature plot for primary testing dataset showing the input features that contributed the most to the machine learning algorithm’s predictions. Abbreviations: pervasive developmental disorders - not otherwise specified (PDD-NOS), restrictive and repetitive behavior (RRB), Social Communication Questionnaire (SCQ). Figure created using Seaborn and Matplotlib in Python

In the primary testing dataset, the top three features impacting the MLA’s performance were SCQ (total score and RRB) and age at which the SCQ was given (Fig. 4). Similarly, the top three features in the supplementary testing dataset included two questions from the ADI-R (social and RRB) and age (demographic) [Online Resource Supplementary Fig. 5].

**Fig. 5:**
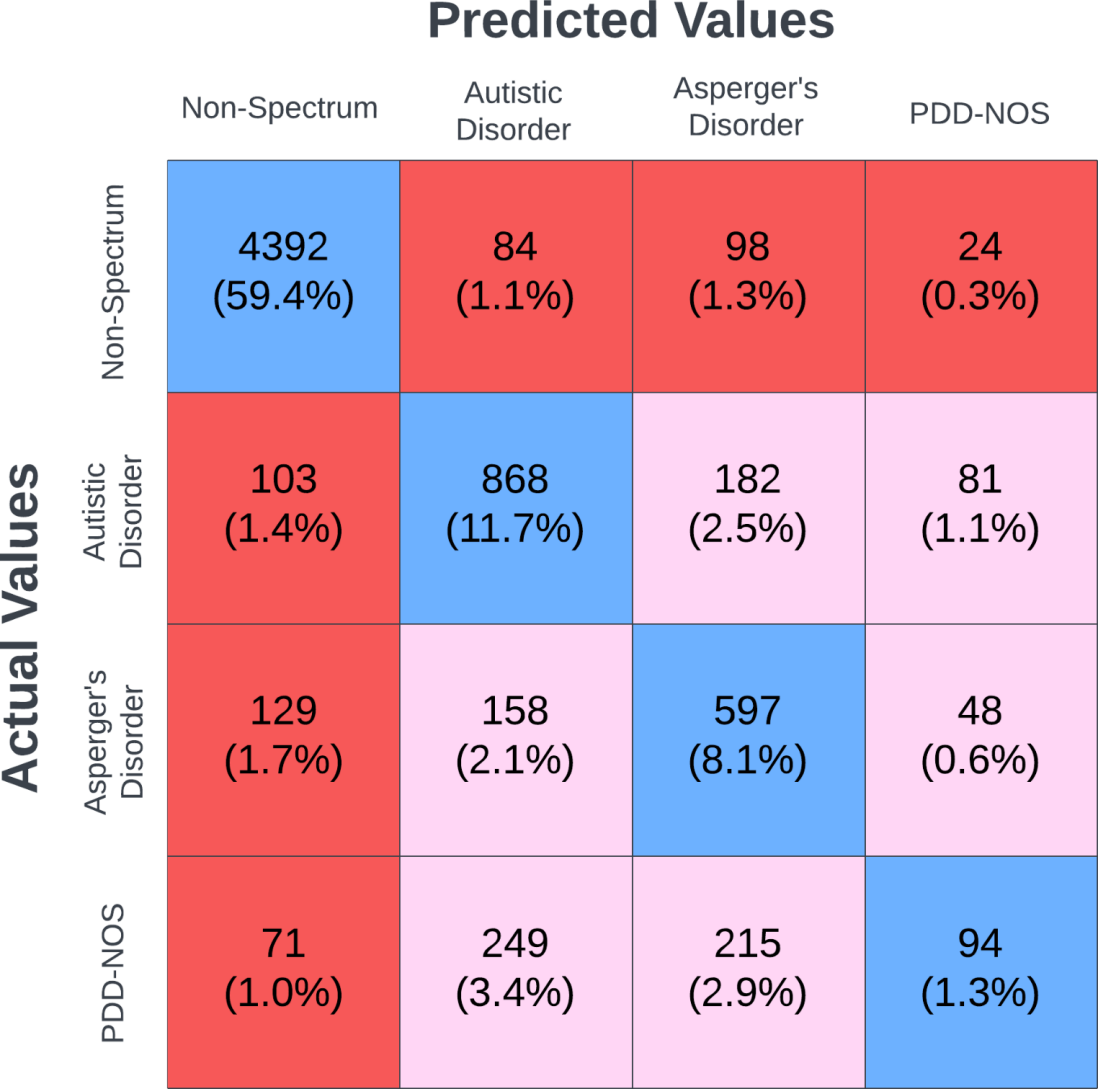
Confusion matrix showing machine learning model multi-classifier outputs for Simons Foundation Powering Autism Research for Knowledge (SPARK) dataset for all classifications. Cells shaded in blue indicate correct predictions for each of the four classes and would be considered a full true positive or full true negative under a binary classification scheme. Cells shaded in red indicate a ground truth non-spectrum individual receiving a prediction to be classified as having autistic disorder, Asperger’s disorder, or PDD-NOS; or alternatively an individual having the ground truth of autistic disorder, Asperger’s disorder, or PDD-NOS receiving a prediction to be classified as non-spectrum. These red cells would be considered full false positives or full false negatives under a binary classification scheme. Pink cells indicate those individuals correctly identified as having a disorder on the autism spectrum, but were misidentified as having the wrong disorder on the autism spectrum (e.g., an individual having the ground truth of Asperger’s disorder being classified as having autistic disorder). Figure created using Lucidchart

The machine learning model was additionally trained for multi-class classification of four classes: autistic disorder, Asperger’s disorder, PDD-NOS, and non-spectrum. The datasets were split similarly as for the binary classification: random split, with 80% of the data in the training dataset and the remaining 20% of the data in the testing dataset, where the training and testing datasets remained completely independent of each other. Training for the multi-class model was performed as described for the binary classifier model. Figure 5 displays the confusion matrix for the multi-class model for the primary testing dataset, showing the performance of the model for each class. Similarly, the confusion matrix for the multi-class model for the supplementary testing dataset is displayed in Online Resource Supplementary Fig. 6, showing the performance of the model for each class. Both the binary and multi-class models were optimized via the hyperparameter optimization described in the Methods section above for the binary classifier model.

Online Resource Supplementary Figs. 7–14 show confusion matrices to provide an additional visual representation of the model’s estimated outputs for both the primary and the supplementary testing datasets for the binary classifiers.

## Discussion

In the present study, we evaluated the performance of an MLA to provide one of four classifications, including autistic disorder, Asperger’s disorder, or PDD-NOS based on the DSM-IV criteria, and one classification indicating non-spectrum. The use of the DSM-IV diagnostic designations was not intended to undermine the validity of the DSM-5 definition of these disorders. Rather, the intent was to contribute to the scientific knowledge about the validity of an ML-based decision support aid to provide support for identifying individuals with ASD. As ASD data derived from DSM-5 criteria become more robust, this research could provide the proof-of-concept basis for a modified MLA to identify patients with ASD per DSM-5 diagnostic criteria. This research may also contribute to the body of field-trial data on the appropriateness of ASD diagnostic criteria and categorization in terms of its ability to accurately and consistently identify individuals with ASD, including those who exhibit atypical characteristics and are thus difficult to categorize. Such data could inform future diagnostic standards. At the patient level, these classifications may prompt a clinician to conduct further evaluation of symptoms and help identify patients in need of interventions as well as tailoring therapeutic strategies for these individuals to ensure that the most effective approaches, in terms of patient outcomes and cost, are utilized (Clancy & Collins, 2010). Though health insurance coverage for ASD treatments may not be available without a DSM-5 ASD diagnosis, identifying individuals with a DSM-IV disorder falling on the autism spectrum could allow these individuals to access specialized educational services (CDC, 2022). Further, a diagnosis of ASD, regardless of the DSM criteria used, may decrease bullying, peer victimization, and self-harm (e.g., suicide) (Hosseini & Molla, 2023; *Suicidality in Autism*, n.d.; Volkmar et al., 2021). This classification MLA may also contribute to the scientific community broadly, as it can provide evidence for the utility of a cost-effective and personalized ML-based approach to evaluate patients with traits of neurodevelopmental disorders. Our MLA could further inform research by identifying a subset of patients with traits of a neurodevelopmental disorder for whom a diagnosis of ASD per DSM-5 criteria would not apply, which could suggest the need for alternative or auxiliary ASD evaluation strategies in clinical and research settings based on individual traits and trends that the MLA can reveal in the data (Clancy & Collins, 2010).

To our knowledge, few studies have used ML techniques to identify the autism spectrum-related classifications. Al-Hiyali conducted a multi-site study in which a convolutional neural network was used to analyze magnetic resonance imaging data to classify individuals with the same DSM-IV diagnostic designations used within our study (Al-Hiyali et al., 2021). Accuracy was acceptable, ranging from 81.7% to 89.2% (Al-Hiyali et al., 2021). Performance of our MLAs is also favorable, with all predictions performing substantially better than baseline (primary testing dataset AUROC range: 0.863–0.980; supplementary testing dataset AUROC range: 0.904–0.969). Our MLAs also displayed favorable values for sensitivity, specificity, PPV, and NPV for all classifications and for each of the datasets (i.e., primary testing dataset and supplementary testing dataset). Of the four classifications in the primary testing dataset, non-spectrum was the most prevalent, followed by autistic disorder and Asperger’s disorder. In the primary testing dataset, language disorder was the most prevalent comorbidity for individuals classified as having autistic disorder (64.5%) and individuals classified as having PDD-NOS (52.2%). Developmental language disorders are a known feature of neurodevelopmental disorders generally, and ASD specifically, as indicated in research and by the DSM-5 criteria (*Autism Diagnosis Criteria*, n.d.; Georgiou and Spanoudis, 2021). In the primary testing dataset, individuals classified as having Asperger’s disorder had the highest prevalence of co-occurring ADHD (55.7%). This was followed in the primary dataset by individuals with PDD-NOS (47.3%) and autistic disorder (32.1%). Overall, these findings are consistent with studies estimating that ADHD co-occurs in 50–70% of individuals with ASD (Hours et al., 2022). Males were more frequently classified as having one of the three disorders (i.e., autistic disorder, Asperger’s disorder, PDD-NOS) than females. However, there were more males included in the dataset than females, therefore, this observation is relative to the number of male and female study participants. While there is no definitive explanation for a higher ASD prevalence in males, some research has suggested that the origin of these differences may be genetic mutations that differ between the sexes or social gender stereotypes that impact diagnosis (Gockley et al., 2015; Jacquemont et al., 2014). In the supplementary testing dataset, ADHD was the most prevalent comorbidity in all three DSM-IV classifications of disorders on the autism spectrum (11.2% for autistic disorder, 32.4% for Asperger’s disorder, 20% for PDD-NOS). These variations in prevalence of comorbidities may be attributed to the difference in size of each of the datasets, wherein the primary dataset analyzed information from 36,965 individuals and the supplementary dataset analyzed information from 1,595 individuals.

For the SPARK dataset, the model correctly classified 5,951 individuals (80.5% of the primary testing dataset). Among the 1,236 individuals with a DSM-IV disorder on the autism spectrum (16.7% of the primary testing dataset) who were misclassified by the model, 303 individuals were incorrectly classified as non-spectrum and 933 were incorrectly classified as having one of the other two DSM-IV disorders on the autism spectrum. Among the 4,598 individuals identified as non-spectrum in the primary testing dataset, the model incorrectly classified only 206 individuals (4.5% of the individuals identified as non-spectrum in the primary testing dataset) with a DSM-IV disorder on the autism spectrum (Fig. 5). Similarly, for the ABIDE dataset, the model correctly classified 262 individuals (82.1% of the supplementary testing dataset). Among the 53 individuals with a DSM-IV disorder on the autism spectrum (16.6% of the supplementary testing dataset) who were misclassified by the model, 22 individuals were incorrectly classified as non-spectrum and 31 were incorrectly classified as having one of the other two DSM-IV disorders on the autism spectrum. Among the 176 individuals who were classified as non-spectrum in the supplementary testing dataset, the model correctly identified 172 as non-spectrum and only misclassified 4 individuals as having a DSM-IV disorder on the autism spectrum (1.3% of the non-spectrum individuals) [Online Resource Supplementary Fig. 6]. Of all of the misclassifications of individuals who had a ground truth of a DSM-IV disorder on the autism spectrum, the majority fell within another of the three classifications for which we evaluated (75.5% in the primary testing dataset and 58.5% in the supplementary testing dataset). These results demonstrate that the MLA achieved relatively high prediction accuracy within both datasets, with the majority of individuals being correctly classified. Further, because most misclassifications for individuals with a DSM-IV disorder on the autism spectrum occurred within those three disorders (i.e., individuals were misclassified as having autistic disorder, Asperger’s disorder, or PDD-NOS), the algorithm may still provide a clinical benefit, as it would ideally prompt further evaluation under DSM-5.

The multi-class classifier models (Fig. 5 and Online Resource Supplementary Fig. 6) are an extension of the binary classification of DSM-IV disorders on the autism spectrum, reflecting the ability of the models to not just classify DSM-IV disorders on the autism spectrum, but also provide more detailed insight into the specific disorder type an individual may have, thus enabling a provider to further specialized treatment or guidance for each individual. In addition, and as detailed above, the majority of misclassifications, in both the primary and supplemental testing datasets, are still correctly classified in a binary classification scheme (DSM-IV disorders on the autism spectrum vs. non-spectrum) as these particular individuals are classified as having a different DSM-IV disorder, but still fall within a diagnosis of autism spectrum. In the primary testing dataset, 6,884 (93.1%) of individuals are correctly classified under a binary scheme. In the supplementary testing dataset, 293 (91.8%) of individuals are correctly classified under a binary scheme. Therefore, the models presented excel at general diagnosis of autism spectrum disorders with a further benefit of offering strong insight to providers on the specific type of disorder an individual may have.

In the primary and supplementary datasets, the top three features contributing to the MLA’s predictions included data from the SCQ and ADI-R questionnaires and age information. This feature overlap across different datasets may indicate the possibility of developing a future MLA which is tailored to a different or broader input availability. RRBs as a significant feature in both datasets reflect that this distinctive set of symptoms has been associated with disorders on the autism spectrum since these disorders were first identified, enough to warrant carrying this forward from DSM-IV to DSM-5 (Iversen & Lewis, 2021; Mahjouri & Lord, 2012). Kim et al. conducted a multi-cohort study of pediatric patients aged ≤ 56 months with typical developmental patterns, patients designated as non-spectrum, or patients with a diagnosis of autistic disorder or PDD-NOS (S. H. Kim & Lord, 2010). Patients with autism had a higher prevalence of RRBs that was statistically significant when compared to patients who displayed developmental behaviors on par with age expectations or were non-spectrum (S. H. Kim & Lord, 2010). With regards to age, several studies have indicated that the worldwide mean age of diagnosis for disorders on the autism spectrum is between 3.2 and 10 years of age (Daniels & Mandell, 2014; van’t Hof et al., 2021). Our findings regarding feature importance are consistent with literature, which we believe adds to the confidence of the model’s predictions.

Other examples of ML employed in ASD diagnosis or risk stratification by other research groups rely on more invasive and/or intensive inputs to make predictions, such as using magnetic resonance imaging or analysis of eye movements to identify atypical patterns when an individual is scanning faces (Al-Hiyali et al., 2021; Eslami et al., 2021; Liu et al., 2016; Santana et al., 2022). In contrast, our MLA demonstrates that DSM-IV disorders on the autism spectrum (i.e., autistic disorder, Asperger’s disorder, and PDD-NOS) can be classified using minimal inputs from EHR data and with no disruption to the clinical workflow. Rather, the MLA receives an individual’s data encompassing medical records, patient demographics, and diagnostic assessments from a database or manually input into the software, analyzes each individual’s data with the MLA to provide a classification as the output, and alerts a clinician that an individual has an increased likelihood of having autistic disorder, Asperger’s disorder, or PDD-NOS; or is classified as likely non-spectrum. Such a tool may be ideal for use in pediatric primary care settings to screen patients who present with symptoms of a neurodevelopmental disorder but may not have a clear path to an ASD diagnosis per DSM-5 criteria, which can prompt further evaluation. We identified only one other study in which ML models for ASD risk assessment used more readily-available data from EHRs (Rahman et al., 2020). In this study, Rahman et al. used EHR data from the parents of newborns to assess the risk of ASD. The three models used (logistic regression, artificial neural networks, and random forest) yielded AUROC values ranging from 0.693 to 0.727 for early prediction of ASD (Rahman et al., 2020). The most important feature contributing to Rahman’s algorithm predictions was the age gap between the mother-father sets (Rahman et al., 2020), whereas the most important features identified in the primary dataset by our MLA was the total score and RRB on the SCQ evaluation. This may be attributed to the data used for assessment, as Rahman’s algorithm analyzed data from the parents of newborns to assess risk. In contrast, our MLA used data from individuals 2 years of age and older, on whom the questionnaire is appropriate to provide assessment of risk of disorders on the autism spectrum. While Rahman’s study indicates promise for the use of EHRs in ASD diagnostics, the robust number of inputs required to make a prediction and modest performance may limit clinical utility in its reported format (Rahman et al., 2020).

### Study Limitations

Our study presents itself with certain limitations. First, the study was conducted using retrospective data, therefore, it is unknown how the MLA would perform in a clinical setting. Future research should involve a prospective clinical analysis to determine the real-world performance of the MLA. Another limitation is that the datasets did not have straightforward overlap of features to allow us to train on one dataset and test on a different dataset. In future research, we plan to focus on increasing the generalizability of the inputs of the MLA beyond the datasets used in this project. Though most metrics demonstrated excellent performance, the PPV for PDD-NOS was 0.391 for SPARK and 0.333 for ABIDE. This may be, in part, due to the relatively low PDD-NOS prevalence within the datasets, and the correlation between prevalence and PPV and NPV - in that a lower prevalence can lead to lower PPV and vice versa (Tenny & Hoffman, 2022). Additionally, this low PPV may be partially attributed to the fact that the MLA disproportionately misclassified individuals with PDD-NOS as having autistic disorder and Asperger’s disorder. Therefore, the low PPV cannot entirely be attributed to low prevalence in the data as a whole and misclassification of these individuals as non-spectrum. Further, the low PPV may be additionally exacerbated by the low prevalence of PDD-NOS relative to the prevalence of the other disorders on the autism spectrum as well.

In terms of comorbidities, the datasets were unbalanced in terms of their representation of comorbidities, particularly ADHD. We expect that this was due to the primary dataset (SPARK) and supplementary (ABIDE) dataset being distinctly different in regards to the type of data they collect. SPARK is comprised of genetic, clinical, and behavioral assessment information. ABIDE is comprised of imaging (functional magnetic resonance image) and phenotypic data. It may therefore be possible that, owing to different recruiting strategies, SPARK and ABIDE enrolled patients with somewhat different characteristics. Additionally, it may be possible that the smaller number of individuals in ABIDE has more statistical variance. Finally, the SPARK dataset did not provide DSM-5 diagnoses data for any of the individuals that were filtered into the primary dataset, and thus, we could not analyze the performance of our model in light of the current standard of practice. However, it should be noted that, within the supplementary dataset derived from ABIDE, a total of 103 individuals had a diagnosis under both DSM-IV and DSM-5, i.e., these 103 individuals had been diagnosed as having a disorder on the autism spectrum under DSM-IV, and were also diagnosed with ASD under DSM-5. Out of these 103 individuals, 20 were randomly placed into the supplementary testing dataset and our prediction algorithm was able to accurately classify all of these 20 individuals as having one of the disorders on the autism spectrum (i.e., autistic disorder, Asperger’s disorder, or PDD-NOS). Future research should examine concordance between the MLA’s identification of a DSM-IV diagnosis of a disorder of the autism spectrum and the DSM-5-defined diagnoses of ASD, and should evaluate the MLA for individuals diagnosed with ASD based on DSM-5 criteria. Future research may also incorporate International Classification of Diseases (ICD) codes to evaluate concordance between ICD and DSM codes for identifying ASD (Wilson et al., 2013). Such an investigational strategy would also allow us to integrate diverse socio-demographic sub-groups than what is currently represented in epidemiological research, as the use of ICD codes for identification of disorders and diseases is largely limited to private healthcare systems. This may further allow us to integrate results from our data analysis using ICD codes with data analysis using DSM diagnoses and thereby contribute to new or existing datasets.

## Conclusions

This research has demonstrated the potential value of ML to identify the specific classifications in which individuals fall on the autism spectrum per DSM-IV for the purpose of guiding clinical practice. This tool may enable clinicians to identify individuals who face an unclear or borderline diagnosis based on the current DSM-5 diagnostic criteria and should prompt deeper or specialized evaluation in these cases. Further, this tool may support clinicians during the diagnostic process by utilizing DSM-IV classification for disorders on the autism spectrum to assess symptoms and help identify patients that may be eligible for a DSM-5 diagnosis of ASD. Reducing or eliminating the challenges in reaching an ASD diagnosis is of vital importance given the complexity of the current ASD diagnostic process and the potential implications of missed diagnosis. For example, it has been posited that adults who receive a late or missed diagnosis are at a high risk for suicide attempts, which some research suggests occurs more frequently with females, particularly when they do not demonstrate an intellectual deficit (Fusar-Poli et al., 2022). Consequently, the earlier a diagnosis of ASD can be made, the better the prognosis and overall quality of life for the affected individual.

## Electronic Supplementary Material

Below is the link to the electronic supplementary material.

Supplementary Material 1

## Footnotes

1 We choose to use the term Asperger’s Disorder in this paper in order to be consistent with DSM-IV terminology, but acknowledge the concerns raised about this term (Sheffer, n.d.; Singh, 2011).

2 For some categories in our results (e.g., comorbidities), percentages do not add up to 100%. This is attributed to overlapping features.
