# Supplementary material for: Machine Learning Differentiation of Autism Spectrum Sub-Classifications
Source: J Autism Dev Disord. 2023 Sep 26;54(11):4216–31. doi: 10.1007/s10803-023-06121-4 (PMC11461775; doi:10.1007/s10803-023-06121-4)
Supplement: Supplementary file 1 — Supplementary Material 1 [file 10803_2023_6121_MOESM1_ESM.docx]

**Supplementary Material**


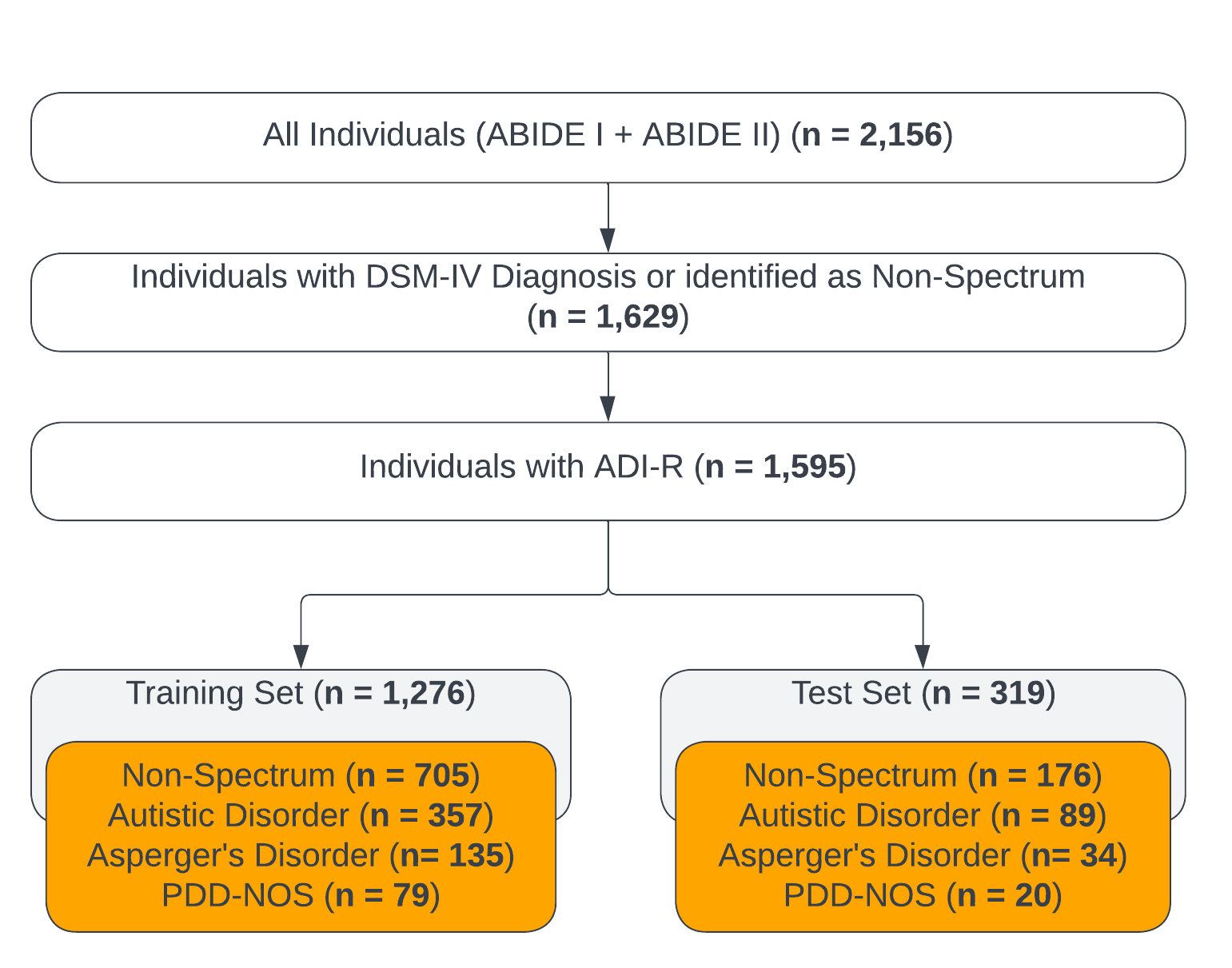


**Supplementary Fig. 1** Attrition chart for ABIDE dataset. Within our supplemental dataset, we included individuals with a DSM-IV diagnosis of a disorder on the autism spectrum and those identified as non-spectrum. The individuals with a DSM-IV diagnosis were also required to have an ADI-R score. The resulting primary dataset was split 80/20 into training and testing datasets, respectively. The training and testing datasets remained completely independent of each other. Abbreviations: Diagnostic and Statistical Manual of Mental Disorders, 4th Edition (DSM-IV), Autism Brain Imaging Data Exchange (ABIDE), Autism Diagnostic Interview-Revised (ADI-R), pervasive developmental disorder - not otherwise specified (PDD-NOS). Figure created using Lucidchart


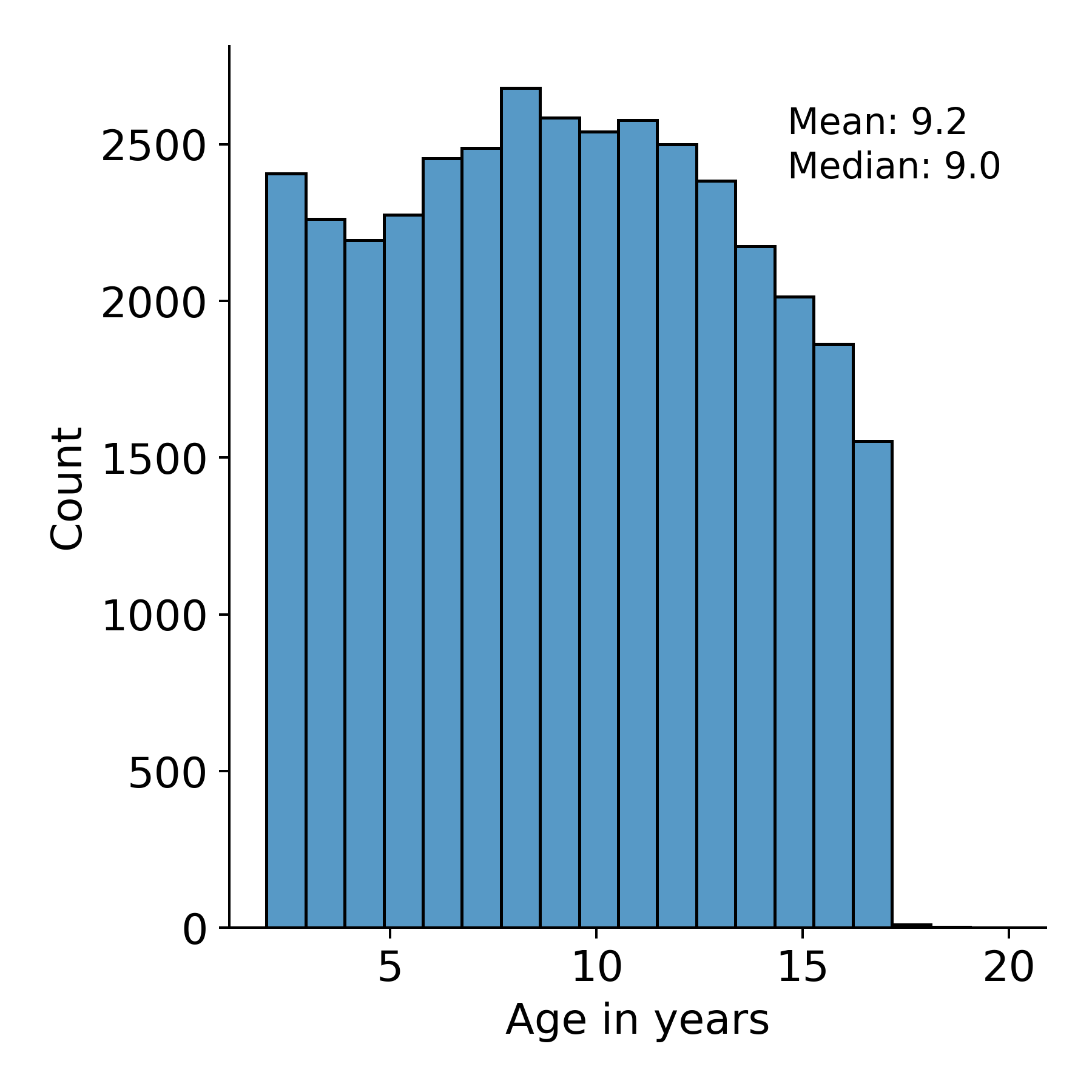


**Supplementary Fig. 2** Mean and median of age in Simons Foundation Powering Autism Research for Knowledge (SPARK) testing and training datasets. Study participants were in the 2-19 years old age range. Figure created using Seaborn and Matplotlib in Python


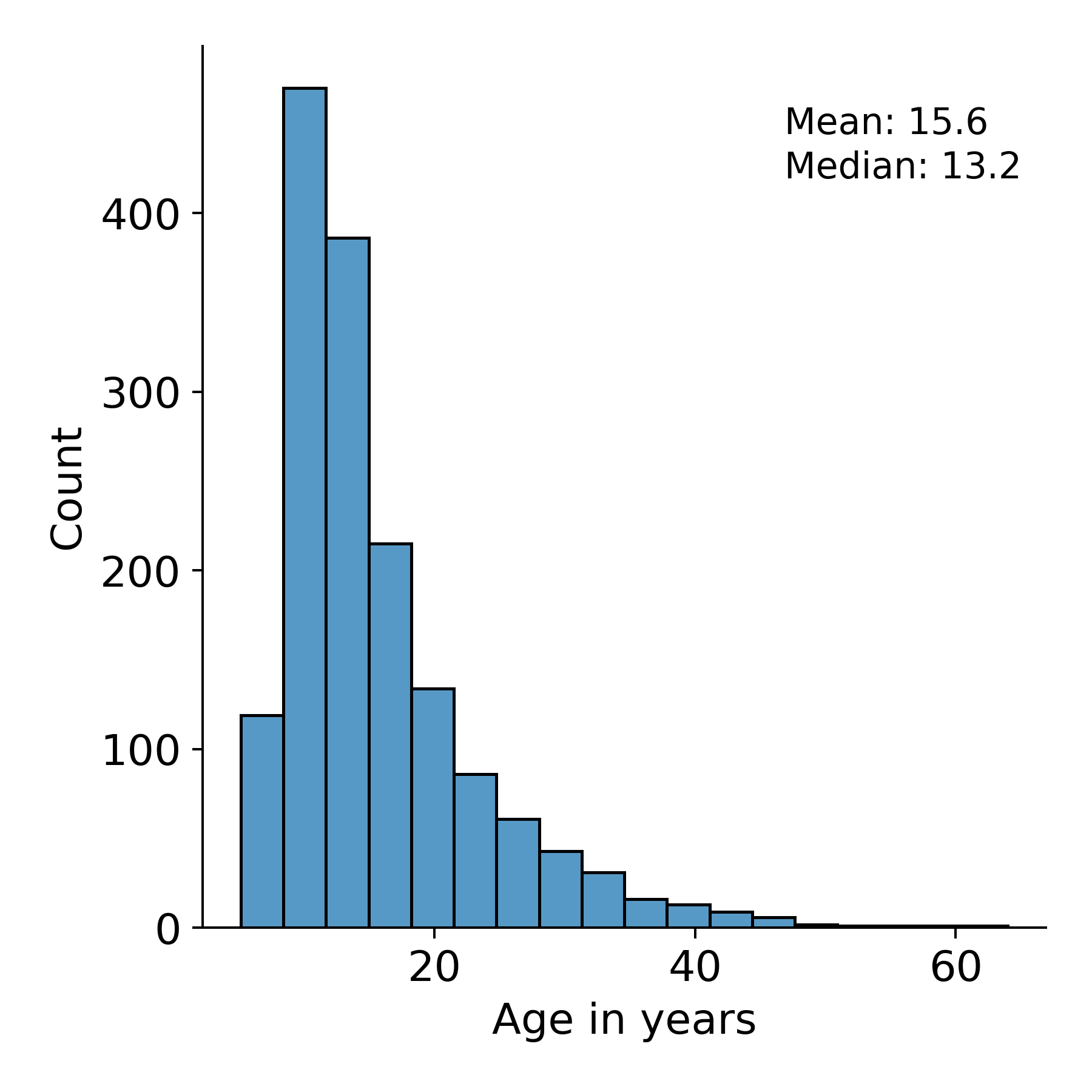


**Supplementary Fig. 3** Mean and median of age in Autism Brain Imaging Data Exchange (ABIDE) testing and training datasets. Participants in this dataset had an age range of 5-64 years old. Figure created using Seaborn and Matplotlib in Python

**Supplementary Table 1.** Optimal hyperparameters for our Machine Learning Algorithms (MLAs) obtained after 5-fold cross validation grid search.

| Hyperparameters | SPARK | ABIDE |
| --- | --- | --- |
| n_estimators | 350 | 100 |
| max_depth | 4 | 5 |
| learning_rate | 0.1 | 0.1 |
| reg_alpha | 0.5 | 1 |
| reg_lambda | 0.8 | 2 |

**Supplementary Table 2.** Data used from the supplementary dataset to generate algorithm outputs. Inputs included demographic information, assessment data, current medication status, comorbidities, and handedness. Abbreviations: machine learning algorithm (MLA), Autism Diagnostic Interview-Revised (ADI-R), autism spectrum disorder (ASD), intelligence quotient (IQ), full scale intelligence quotient (FIQ), verbal intelligence quotient (VIQ), performance intelligence quotient (PIQ), attention deficit hyperactivity disorder (ADHD), obsessive-compulsive disorder (OCD), oppositional defiant disorder (ODD).

| **MLA Inputs** | |
| --- | --- |
| **Demographics**  Age  Sex | **Comorbidities**  Attention Deficit Hyperactivity Disorder (ADHD)  Oppositional Defiant Disorder (ODD)  Obsessive-Compulsive Disorder (OCD)  Anxiety/Generalized Anxiety Disorder  Phobia |
| **Assessment Data**   - Intelligence Quotient (IQ):   - Full-scale IQ (FIQ)   - Verbal IQ (VIQ)   - Performance IQ (PIQ) - Autism Diagnostic Interview-Revised (ADI-R)   - Social Total   - Verbal Total   - Restricted, Repetitive Behaviors Total   - Onset Total | **Handedness**   - Left - Right - Ambidextrous |
| Current Medication Status |  |

**Supplementary Table 3.** Demographic table using Simons Foundation Powering Autism Research for Knowledge (SPARK) testing dataset showing the breakdown of individuals in each classification by age, gender, and race/ethnicity. This table also shows the comorbidities present within each classification. Abbreviations: attention deficit hyperactivity disorder (ADHD), obsessive-compulsive disorder (OCD), oppositional defiant disorder (ODD), pervasive developmental disorder - not otherwise specified (PDD-NOS).

| **Category** | **Demographics** | **TESTING DATASET (N = 7,393)** | | | |
| --- | --- | --- | --- | --- | --- |
|  |  | **Non-Spectrum (N = 4,598)** | **Autistic Disorder  (N = 1,249)** | **Asperger's Disorder  (N = 934)** | **PDD-NOS (N = 634)** |
| **Age (years)** | 2-4 | 738 (16.1%) | 166 (13.3%) | 17 (1.8%) | 20 (3.1%) |
|  | 4-13 | 2847 (61.9%) | 797 (63.8%) | 510 (54.6%) | 363 (57.3%) |
|  | 13-20 | 1013 (22.0%) | 286 (22.9%) | 407 (43.6%) | 251 (39.6%) |
| **Gender** | Male | 2222 (48.3%) | 937 (75.0%) | 701 (75.1%) | 490 (77.3%) |
|  | Female | 2376 (51.7%) | 312 (25.0%) | 233 (24.9%) | 144 (22.7%) |
| **Race/**  **Ethnicity** | White and Non-Hispanic | 1170 (25.4%) | 593 (63.5%) | 638 (51.1%) | 356 (56.2%) |
|  | Black and Non-Hispanic | 112 (2.4%) | 34 (3.6%) | 104 (8.3%) | 34 (5.4%) |
|  | Asian and Non-Hispanic | 82 (1.8%) | 16 (1.7%) | 41 (3.3%) | 13 (2.1%) |
|  | Hispanic | 316 (6.9%) | 97 (10.4%) | 221 (17.7%) | 89 (14.0%) |
|  | Native American | 37 (0.8%) | 27 (2.9%) | 19 (1.5%) | 10 (1.6%) |
|  | Native Hawaiian | 8 (0.2%) | 11 (1.2%) | 10 (0.8%) | 1 (0.2%) |
|  | Others | 15 (0.3%) | 8 (0.9%) | 8 (0.6%) | 6 (0.9%) |
|  | Unknown | 2858 (62.2%) | 148 (15.8%) | 208 (16.7%) | 125 (19.7%) |
| **Comorbidities** | ADHD | 701 (15.2%) | 401 (32.1%) | 520 (55.7%) | 300 (47.3%) |
|  | ODD | 98 (2.1%) | 84 (6.7%) | 136 (14.6%) | 74 (11.7%) |
|  | OCD | 59 (1.3%) | 110 (8.8%) | 144 (15.4%) | 55 (8.7%) |
|  | Anxiety | 387 (8.4%) | 181 (14.5%) | 353 (37.8%) | 159 (25.1%) |
|  | Language Disorder | 375 (8.2%) | 806 (64.5%) | 198 (21.2%) | 331 (52.2%) |

Note: For some categories in our results (e.g., comorbidities), percentages do not add up to 100%. This is attributed to overlapping features.

**Supplementary Table 4.** Demographic table using Autism Brain Imaging Data Exchange (ABIDE) training dataset showing the breakdown of individuals in each classification by age and gender. This table also shows the comorbidities present within each classification and intelligence quotient scores. Abbreviations: full scale intelligence quotient (FIQ), verbal intelligence quotient (VIQ), performance intelligence quotient (PIQ), attention deficit hyperactivity disorder (ADHD), obsessive-compulsive disorder (OCD), oppositional defiant disorder (ODD), pervasive developmental disorder - not otherwise specified (PDD-NOS).

| **Category** | **Demographics** | **TRAINING DATASET (N = 1,276)** | | | |
| --- | --- | --- | --- | --- | --- |
|  |  | **Non-Spectrum  (N = 705)** | **Autistic Disorder  (N = 357)** | **Asperger's Disorder  (N = 135)** | **PDD-NOS  (N = 79)** |
| **Age (years)** | 5-13 | 339 (48.1%) | 147 (41.2%) | 63 (46.7%) | 58 (73.4%) |
|  | 13-20 | 204 (28.9%) | 143 (40.1%) | 29 (21.5%) | 13 (16.5%) |
|  | 20-64 | 162 (23.0%) | 67 (18.8%) | 43 (31.9%) | 8 (10.1%) |
| **Gender** | Male | 531 (75.3%) | 313 (87.7%) | 114 (84.4%) | 73 (92.4%) |
|  | Female | 174 (24.7%) | 44 (12.3%) | 21 (15.6%) | 6 (7.6%) |
| **Comorbidities** | ADHD | 0 (0.0%) | 22 (6.2%) | 31 (23.0%) | 26 (32.9%) |
|  | ODD | 0 (0.0%) | 7 (2.0%) | 9 (6.7%) | 5 (6.3%) |
|  | OCD | 0 (0.0%) | 0 (0.0%) | 4 (3.0%) | 2 (2.5%) |
|  | Anxiety | 0 (0.0%) | 7 (2.0%) | 4 (3.0%) | 5 (6.3%) |
|  | Phobias | 8.0 (1.1%) | 8 (2.2%) | 11 (8.1%) | 2 (2.5%) |
| **FIQ** | <70 | 0 (0.0%) | 5 (1.4%) | 0 (0.0%) | 2 (2.5%) |
|  | 70-80 | 7 (1.0%) | 34 (9.5%) | 5 (3.7%) | 6 (7.6%) |
|  | 80-90 | 18 (2.6%) | 50 (14.0%) | 10 (7.4%) | 13 (16.5%) |
|  | 90-100 | 81 (11.5%) | 62 (17.4%) | 23 (17.0%) | 12 (15.2%) |
|  | 100-110 | 181 (25.7%) | 77 (21.6%) | 28 (20.7%) | 12 (15.2%) |
|  | 110-120 | 184 (26.1%) | 54 (15.1%) | 25 (18.5%) | 21 (26.6%) |
|  | 120-130 | 130 (18.4%) | 33 (9.2%) | 23 (17.0%) | 6 (7.6%) |
|  | 130-140 | 43 (6.1%) | 11 (3.1%) | 13 (9.6%) | 4 (5.1%) |
|  | Unknown | 61 (8.6%) | 31 (8.7%) | 8 (5.9%) | 3 (3.8%) |
| **VIQ** | <70 | 0 (0.0%) | 12 (3.4%) | 0 (0.0%) | 2 (2.5%) |
|  | 70-80 | 2 (0.3%) | 27 (7.6%) | 1 (0.7%) | 3 (3.8%) |
|  | 80-90 | 25 (3.5%) | 54 (15.1%) | 6 (4.4%) | 12 (15.2%) |
|  | 90-100 | 75 (10.6%) | 74 (20.7%) | 15 (11.1%) | 13 (16.5%) |
|  | 100-110 | 126 (17.9%) | 70 (19.6%) | 25 (18.5%) | 15 (19.0%) |
|  | 110-120 | 165 (23.4%) | 52 (14.6%) | 24 (17.8%) | 12 (15.2%) |
|  | 120-130 | 123 (17.4%) | 27 (7.6%) | 18 (13.3%) | 11 (13.9%) |
|  | 130-140 | 56 (7.9%) | 16 (4.5%) | 15 (11.1%) | 6 (7.6%) |
|  | Unknown | 133 (18.9%) | 25 (7.0%) | 31 (22.9%) | 5 (6.3%) |
| **PIQ** | <70 | 1 (0.1%) | 9 (2.5%) | 3 (2.2%) | 3 (3.8%) |
|  | 70-80 | 8 (1.1%) | 21 (5.9%) | 4 (3.0%) | 7 (8.9%) |
|  | 80-90 | 34 (4.8%) | 57 (16.0%) | 11 (8.1%) | 12 (15.2%) |
|  | 90-100 | 95 (13.5%) | 54 (15.1%) | 9 (6.7%) | 16 (20.3%) |
|  | 100-110 | 189 (26.8%) | 77 (21.6%) | 30 (22.2%) | 14 (17.7%) |
|  | 110-120 | 162 (23.0%) | 71 (19.9%) | 23 (17.0%) | 11 (13.9%) |
|  | 120-130 | 94 (13.3%) | 34 (9.5%) | 19 (14.1%) | 5 (6.3%) |
|  | 130-140 | 28 (4.0%) | 12 (3.4%) | 5 (3.7%) | 5 (6.3%) |
|  | Unknown | 94 (13.3%) | 22 (6.2%) | 31 (22.9%) | 6 (7.6%) |

Note: For some categories in our results (e.g., comorbidities), percentages do not add up to 100%. This is attributed to overlapping features.

**Supplementary Table 5.** Demographic table using Autism Brain Imaging Data Exchange (ABIDE) testing dataset showing the breakdown of individuals in each classification by age and gender. This table also shows the comorbidities present within each classification and intelligence quotient scores. Abbreviations: full scale intelligence quotient (FIQ), verbal intelligence quotient (VIQ), performance intelligence quotient (PIQ), attention deficit hyperactivity disorder (ADHD), obsessive-compulsive disorder (OCD), oppositional defiant disorder (ODD), pervasive developmental disorder - not otherwise specified (PDD-NOS).

| **Category** | **Demographics** | **TESTING DATASET (N = 319)** | | | |
| --- | --- | --- | --- | --- | --- |
|  |  | **Non-Spectrum  (N = 176)** | **Autistic Disorder  (N = 89)** | **Asperger's Disorder  (N = 34)** | **PDD-NOS  (N = 20)** |
| **Age (years)** | 5-13 | 93 (52.8%) | 35 (39.3%) | 21 (61.8%) | 17 (85.0%) |
|  | 13-20 | 53 (30.1%) | 34 (38.2%) | 9 (26.5%) | 3 (15.0%) |
|  | 20-58 | 30 (17.0%) | 20 (22.5%) | 4 (11.8%) | 0 (0.0%) |
| **Gender** | Male | 138 (78.4%) | 81 (91.0%) | 28 (82.4%) | 16 (80.0%) |
|  | Female | 38 (21.6%) | 8 (9.0%) | 6 (17.6%) | 4 (20.0%) |
| **Comorbidities** | ADHD | 0 (0.0%) | 10 (11.2%) | 11 (32.4%) | 4 (20.0%) |
|  | ODD | 0 (0.0%) | 2 (2.2%) | 4 (11.8%) | 0 (0.0%) |
|  | OCD | 0 (0.0%) | 1 (1.1%) | 1 (2.9%) | 0 (0.0%) |
|  | Anxiety | 0 (0.0%) | 4 (4.5%) | 2 (5.9%) | 2 (10.0%) |
|  | Phobias | 2 (1.1%) | 3 (3.4%) | 8 (23.5%) | 1 (5.0%) |
| **FIQ** | <70 | 0 (0.0%) | 2 (2.2%) | 1 (2.9%) | 1 (5.0%) |
|  | 70-80 | 2 (1.1%) | 5 (5.6%) | 0 (0.0%) | 1 (5.0%) |
|  | 80-90 | 4 (2.3%) | 7 (7.9%) | 3 (8.8%) | 3 (15.0%) |
|  | 90-100 | 25 (14.2%) | 25 (28.1%) | 3 (8.8%) | 4 (20.0%) |
|  | 100-110 | 38 (21.6%) | 16 (18.0%) | 8 (23.5%) | 4 (20.0%) |
|  | 110-120 | 49 (27.8%) | 14 (15.7%) | 11 (32.4%) | 3 (15.0%) |
|  | 120-130 | 26 (14.8%) | 13 (14.6%) | 8 (23.5%) | 0 (0.0%) |
|  | 130-140 | 8 (4.5%) | 3 (3.4%) | 0 (0.0%) | 3 (15.0%) |
|  | Unknown | 24 (13.6%) | 4 (4.5%) | 0 (0.0%) | 1 (5.0%) |
| **VIQ** | <70 | 0 (0.0%) | 3 (3.4%) | 0 (0.0%) | 1 (5.0%) |
|  | 70-80 | 1 (0.6%) | 2 (2.2%) | 0 (0.0%) | 1 (5.0%) |
|  | 80-90 | 10 (5.7%) | 10 (11.2%) | 0 (0.0%) | 1 (5.0%) |
|  | 90-100 | 14 (8.0%) | 21 (23.6%) | 6 (17.6%) | 4 (20.0%) |
|  | 100-110 | 35 (19.9%) | 21 (23.6%) | 5 (14.7%) | 6 (30.0%) |
|  | 110-120 | 39 (22.2%) | 15 (16.9%) | 9 (26.5%) | 3 (15.0%) |
|  | 120-130 | 31 (17.6%) | 11 (12.4%) | 8 (23.5%) | 2 (10.0%) |
|  | 130-140 | 11 (6.2%) | 3 (3.4%) | 2 (5.9%) | 1 (5.0%) |
|  | Unknown | 35 (19.9%) | 3 (3.4%) | 4 (11.8%) | 1 (5.0%) |
| **PIQ** | <70 | 0 (0.0%) | 4 (4.5%) | 1 (2.9%) | 0 (0.0%) |
|  | 70-80 | 4 (2.3%) | 1 (1.1%) | 1 (2.9%) | 3 (15.0%) |
|  | 80-90 | 8 (4.5%) | 12 (13.5%) | 2 (5.9%) | 3 (15.0%) |
|  | 90-100 | 25 (14.2%) | 18 (20.2%) | 2 (5.9%) | 4 (20.0%) |
|  | 100-110 | 47 (26.7%) | 18 (20.2%) | 13 (38.2%) | 2 (10.0%) |
|  | 110-120 | 38 (21.6%) | 19 (21.3%) | 5 (14.7%) | 4 (20.0%) |
|  | 120-130 | 20 (11.4%) | 9 (10.1%) | 4 (11.8%) | 1 (5.0%) |
|  | 130-140 | 6 (3.4%) | 2 (2.2%) | 1 (2.9%) | 3 (15.0%) |
|  | Unknown | 28 (15.9%) | 6 (6.7%) | 5 (14.7%) | 0 (0.0%) |

Note: For some categories in our results (e.g., comorbidities), percentages do not add up to 100%. This is attributed to overlapping features.


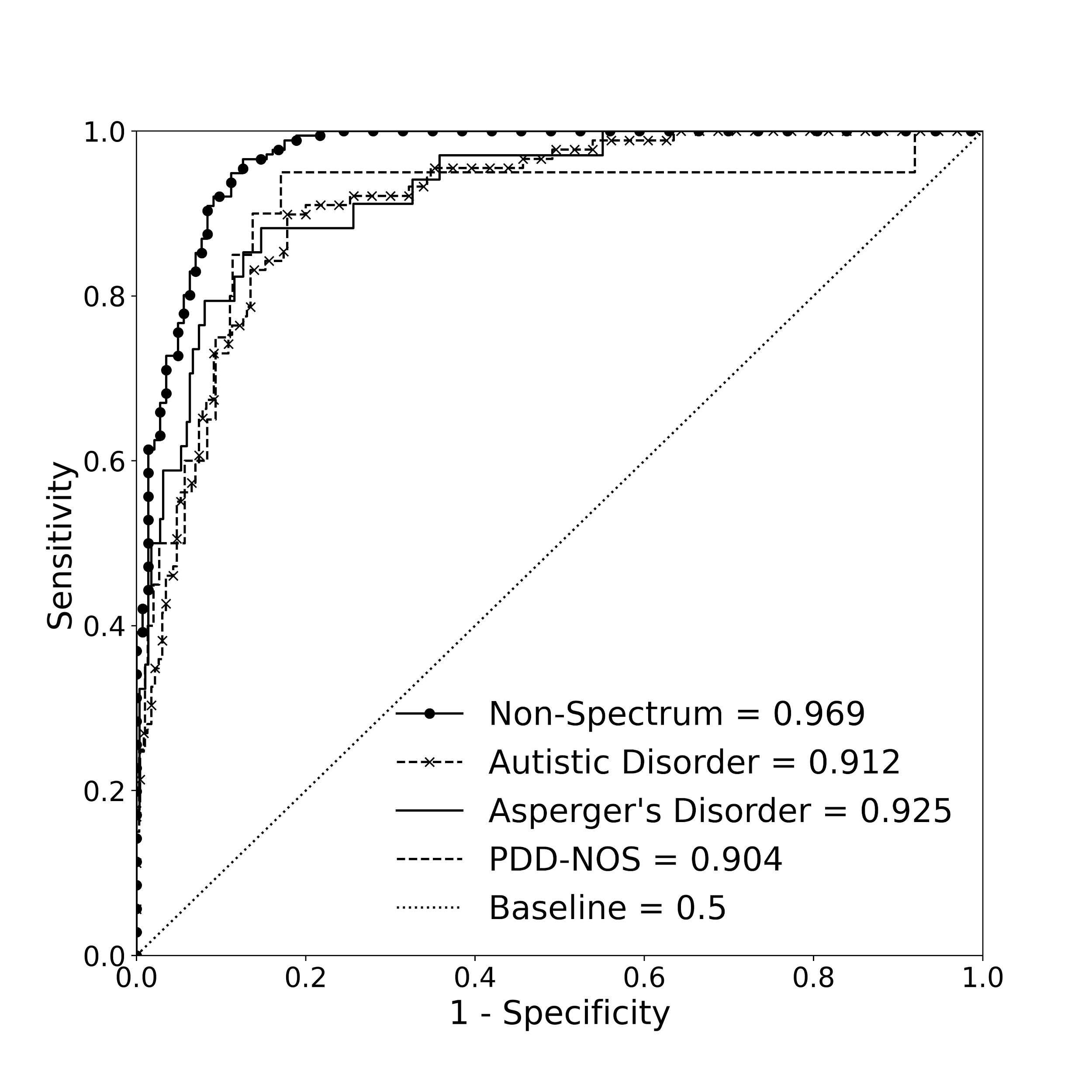


**Supplementary Fig. 4** Area under the receiver operating characteristic curve (AUROC) demonstrating the machine learning algorithm’s (MLA’s) performance for classifying individuals into the four output classifications using Autism Brain Imaging Data Exchange (ABIDE) testing dataset. For all four classifications, the MLA performed better than the baseline. The baseline curve represents a model that is not able to differentiate between classifications at all, effectively equivalent to a random coin-flip. Abbreviation: pervasive developmental disorder - not otherwise specified (PDD-NOS). Figure created using Seaborn and Matplotlib in Python

**Supplementary Table 6.** Area under the receiver operating characteristic curve (AUROC) demonstrating classification performance of the machine learning algorithm in each of the four classifications in the supplementary testing dataset as determined by the AUROC, sensitivity, specificity, positive predictive value, negative predictive value. All metrics include a 95% confidence interval. The prevalence of each classification using the supplementary testing dataset is also shown. Abbreviations: confidence interval (CI), negative predictive value (NPV), positive predictive value (PPV), pervasive developmental disorder - not otherwise specified (PDD-NOS).

|  | **Non-Spectrum** | **Autistic Disorder** | **Asperger’s Disorder** | **PDD-NOS** |
| --- | --- | --- | --- | --- |
| **AUROC  (95% CI*)** | 0.969  (0.951 - 0.984) | 0.912  (0.877 - 0.944) | 0.925  (0.876 - 0.965) | 0.904  (0.798 - 0.971) |
| **Sensitivity  (95% CI)** | 0.852  (0.800 - 0.905) | 0.854  (0.781 - 0.927) | 0.853  (0.734 - 0.972) | 0.850  (0.694 - 1.006) |
| **Specificity  (95% CI)** | 0.930  (0.888 - 0.972) | 0.826  (0.777 - 0.875) | 0.874  (0.835 - 0.912) | 0.886  (0.850 - 0.922) |
| **PPV  (95% CI)** | 0.938  (0.900 - 0.975) | 0.655  (0.569 - 0.742) | 0.446  (0.325 - 0.567) | 0.333  (0.204 - 0.463) |
| **NPV  (95% CI)** | 0.836  (0.779 - 0.894) | 0.936  (0.902 - 0.970) | 0.980  (0.963 - 0.997) | 0.989  (0.976 - 1.001) |
| **Prevalence** | 0.552 | 0.279 | 0.107 | 0.063 |


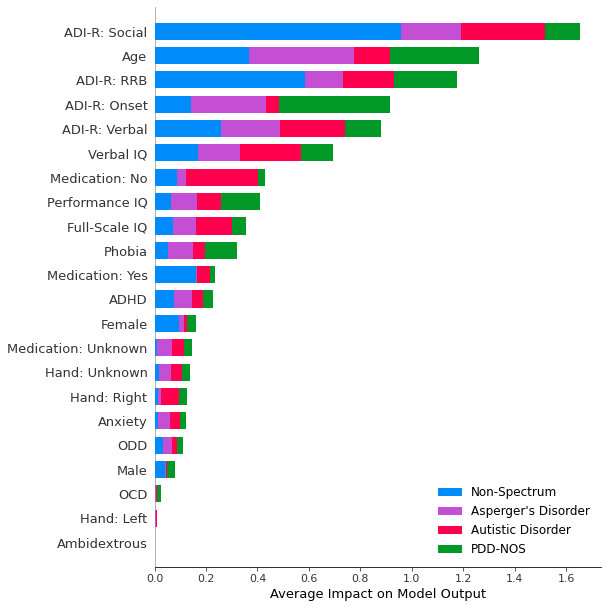


**Supplementary Fig. 5** Feature plot for supplementary testing dataset showing the input features that contributed the most to the machine learning algorithm’s predictions. Abbreviations: Autism Diagnostic Interview - Revised (ADI-R), attention deficit hyperactivity disorder (ADHD), intelligence quotient (IQ), oppositional defiant disorder (ODD), obsessive compulsive disorder (OCD), pervasive developmental disorder - not otherwise specified, restrictive and repetitive behavior (RRB). Figure created using Seaborn and Matplotlib in Python


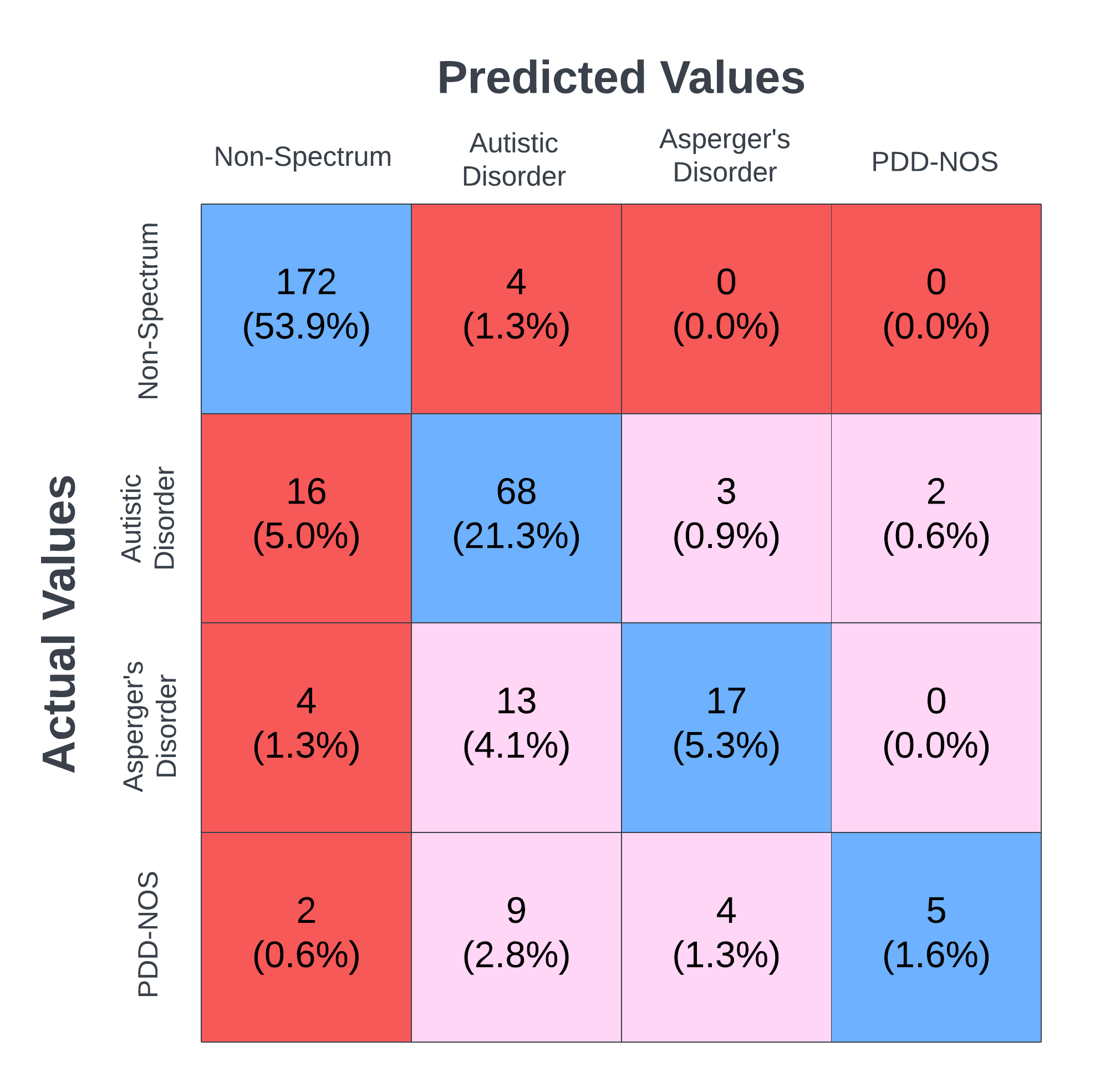


**Supplementary Fig. 6** Confusion matrix showing machine learning model multi-classifier output for Autism Brain Imaging Data Exchange (ABIDE) dataset for all classifications. Figure created using Lucidchart


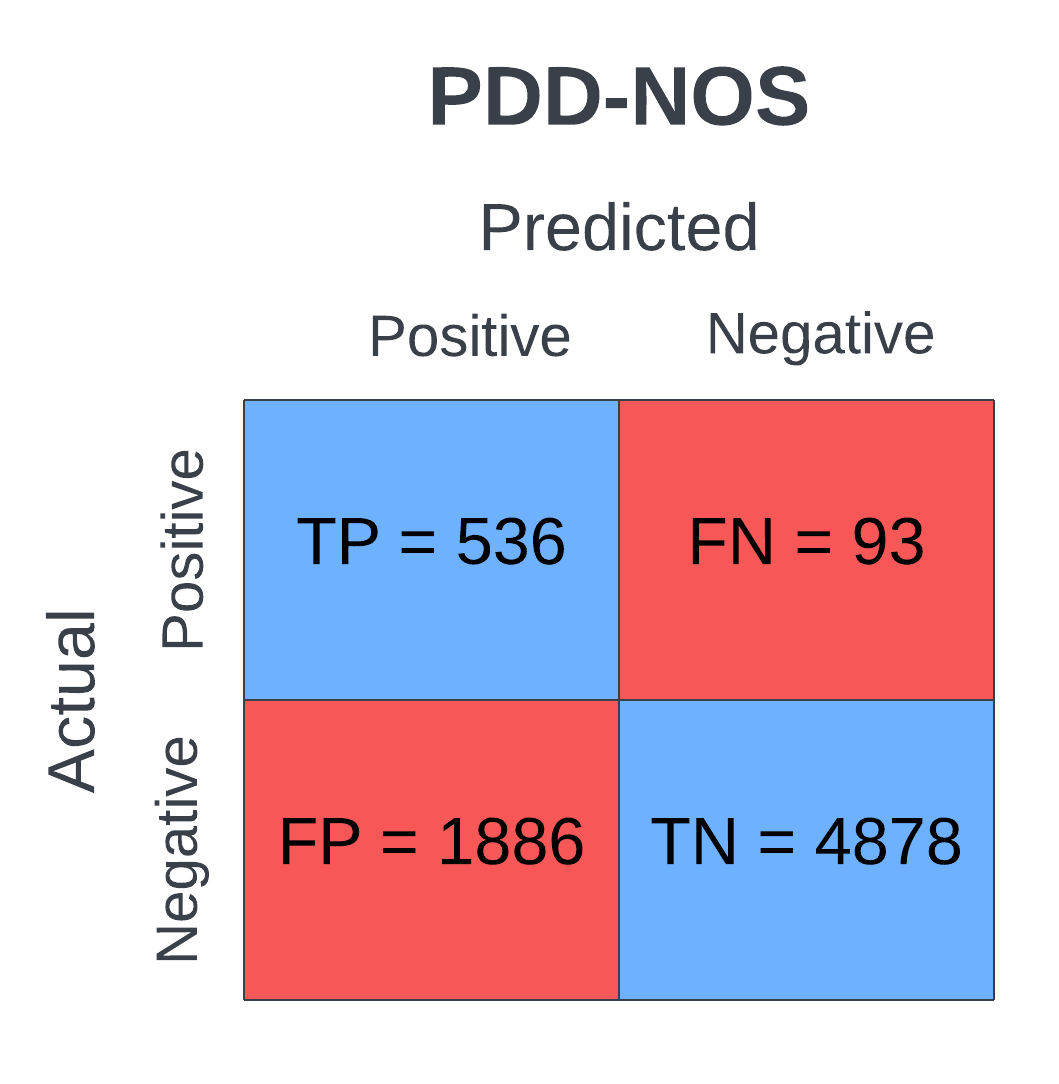


**Supplementary Fig. 7** Confusion matrix showing machine learning model output for Simons Foundation Powering Autism Research for Knowledge (SPARK) dataset for pervasive developmental disorder - not otherwise specified (PDD-NOS). “Positive” is equivalent to PDD-NOS, and “Negative” is equivalent to all other categories combined, i.e., non-spectrum and autistic disorder and Asperger’s disorder. Figure created using Lucidchart


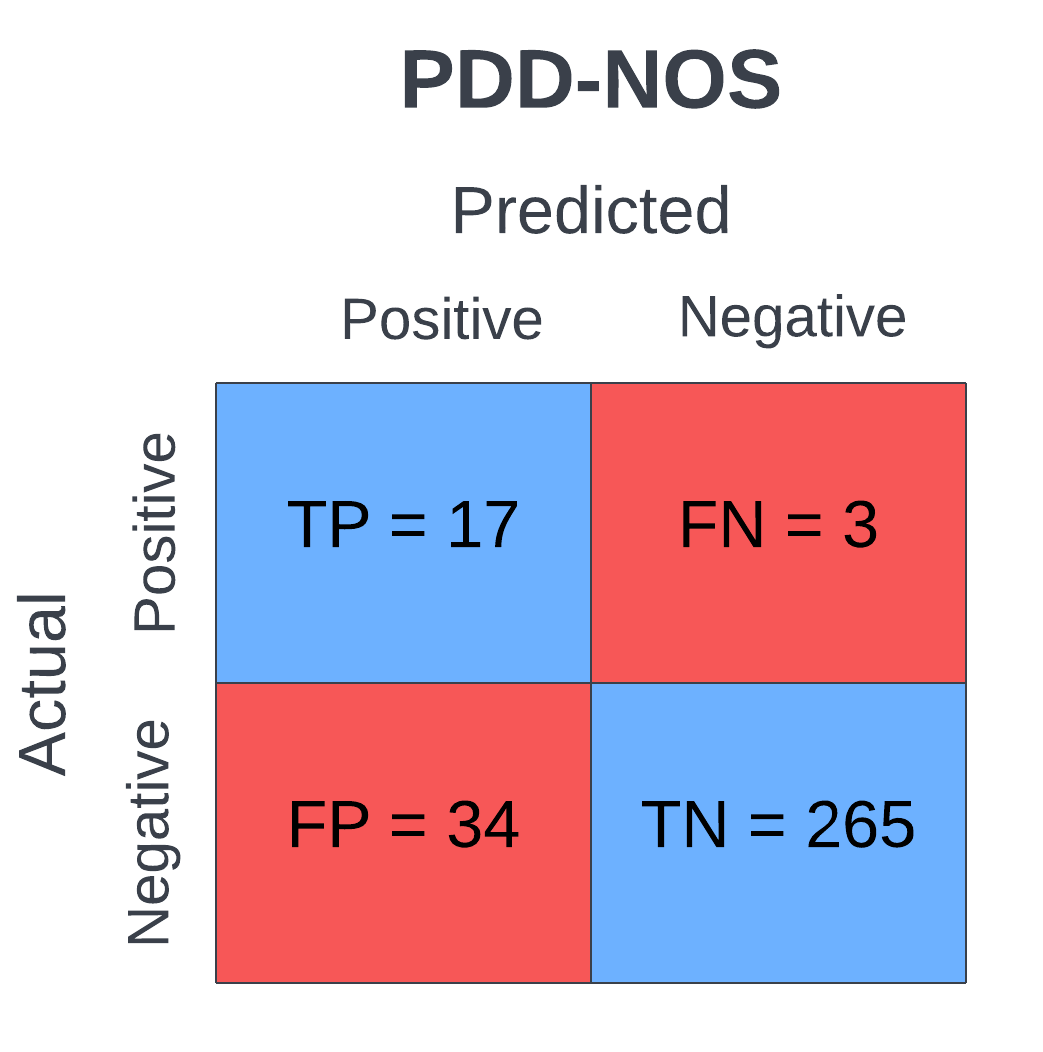


**Supplementary Fig. 8** Confusion matrix showing machine learning model output for Autism Brain Imaging Data Exchange (ABIDE) dataset for pervasive developmental disorder - not otherwise specified (PDD-NOS). “Positive” is equivalent to PDD-NOS, and “Negative” is equivalent to all other categories combined, i.e., non-spectrum and autistic disorder and Asperger’s disorder. Figure created using Lucidchart


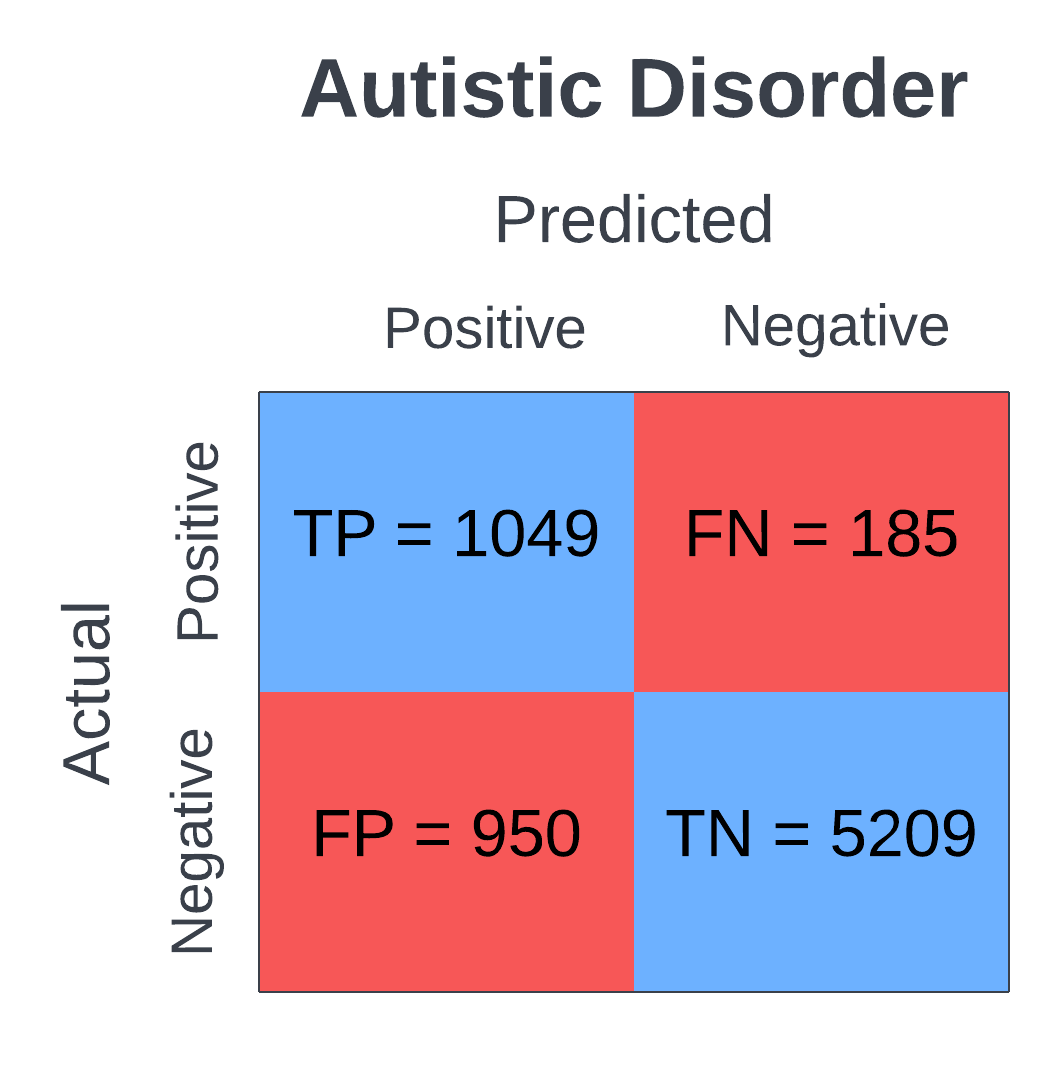


**Supplementary Fig. 9** Confusion matrix showing machine learning model output for Simons Foundation Powering Autism Research for Knowledge (SPARK) dataset for autistic disorder. “Positive” is equivalent to autistic disorder, and “Negative” is equivalent to all other categories combined, i.e., non-spectrum and Asperger’s disorder and pervasive developmental disorder - not otherwise specified. Figure created using Lucidchart


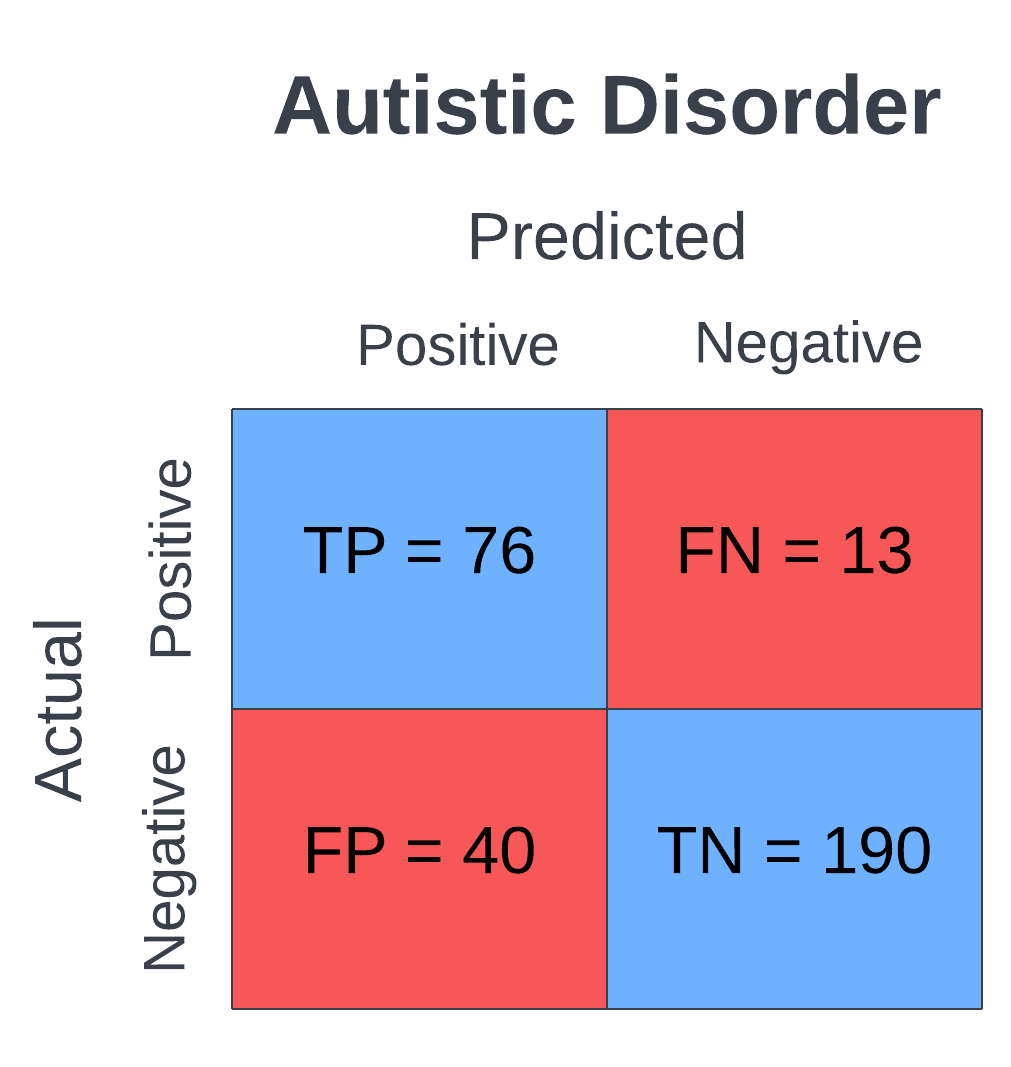


**Supplementary Fig. 10** Confusion matrix showing machine learning model output for Autism Brain Imaging Data Exchange (ABIDE) dataset for autistic disorder. “Positive” is equivalent to autistic disorder, and “Negative” is equivalent to all other categories combined, i.e., non-spectrum and Asperger’s disorder and pervasive developmental disorder - not otherwise specified. Figure created using Lucidchart


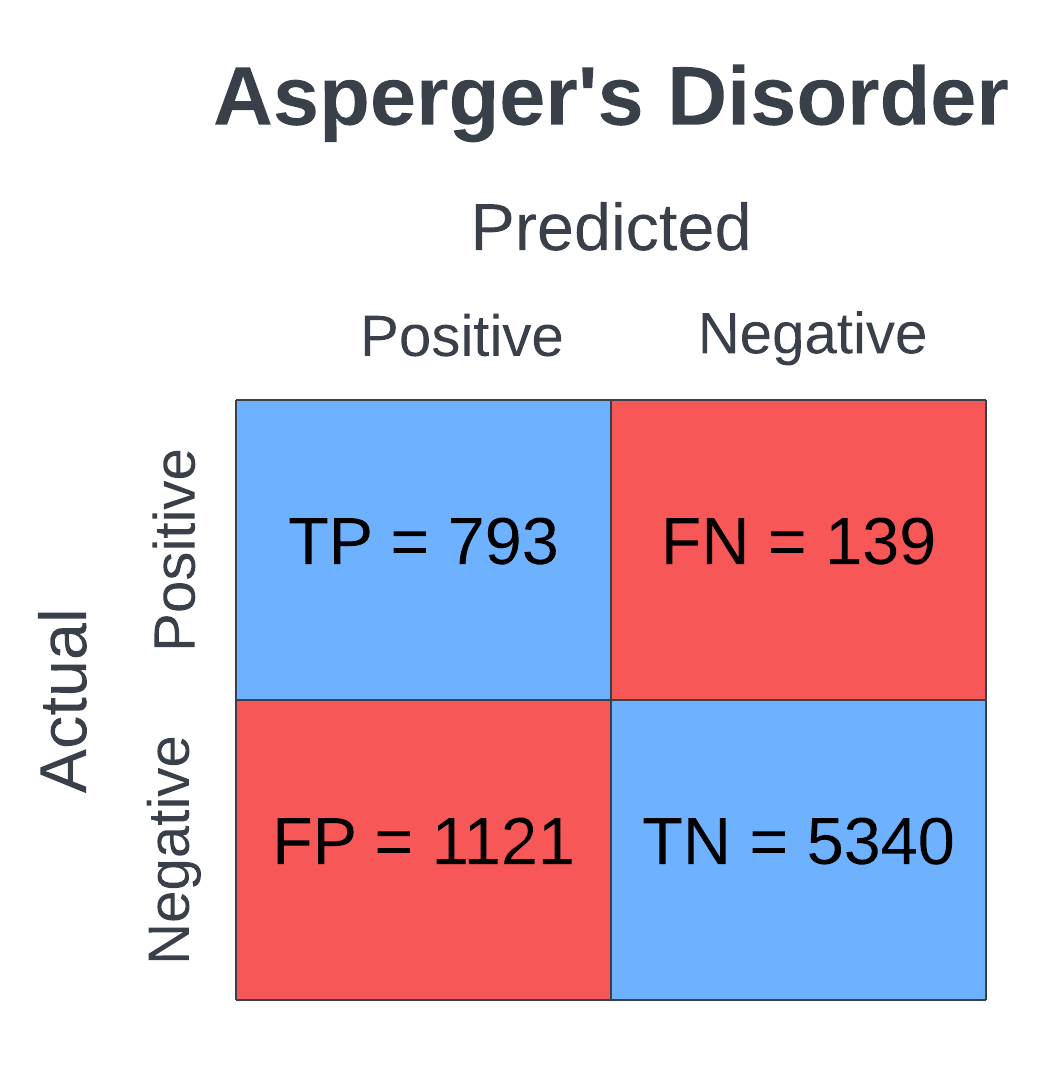


**Supplementary Fig. 11** Confusion matrix showing machine learning model output for Simons Foundation Powering Autism Research for Knowledge (SPARK) dataset for Asperger’s disorder. “Positive” is equivalent to Asperger’s disorder, and “Negative” is equivalent to all other categories combined, i.e., non-spectrum and autistic disorder and pervasive developmental disorder - not otherwise specified. Figure created using Lucidchart


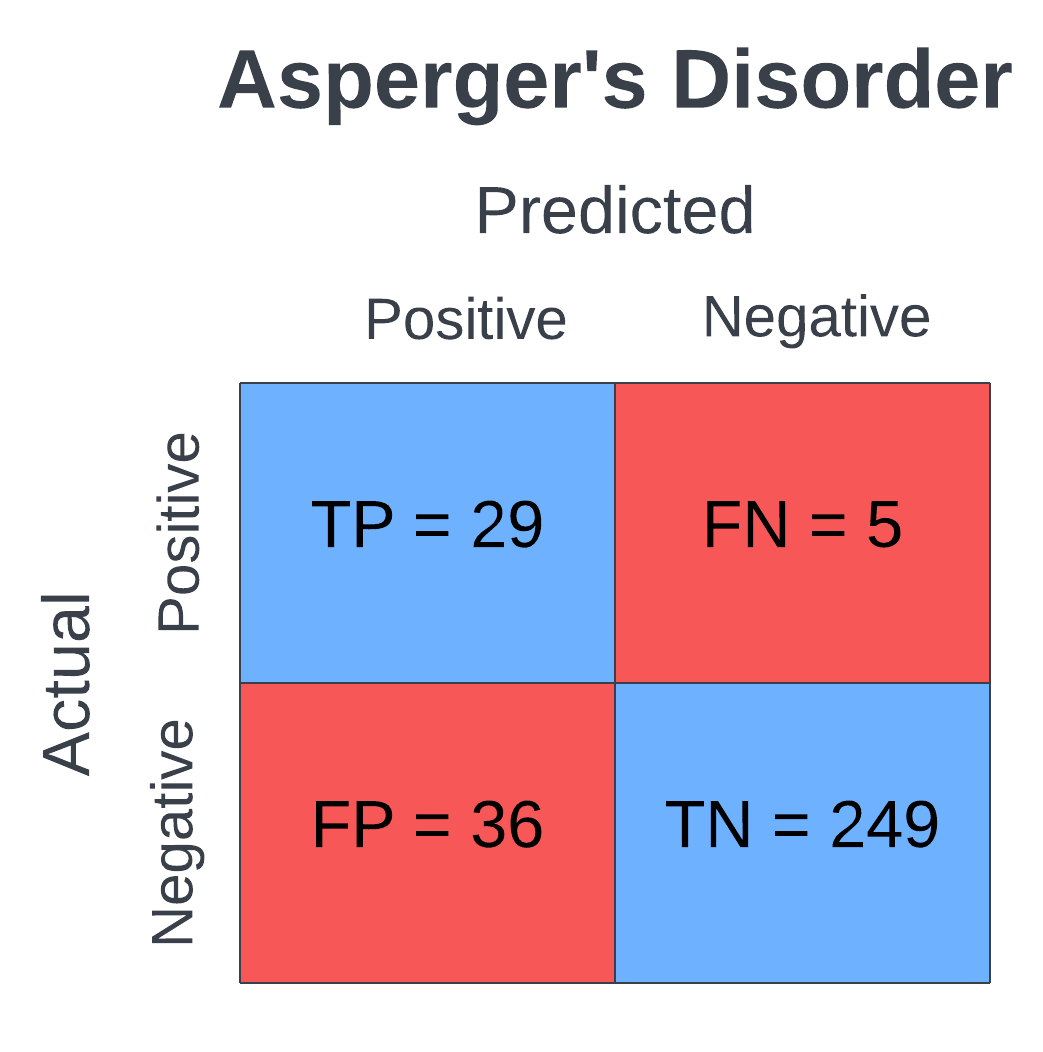


**Supplementary Fig. 12** Confusion matrix showing machine learning model output for Autism Brain Imaging Data Exchange (ABIDE) dataset for Asperger’s disorder. “Positive” is equivalent to Asperger’s disorder, and “Negative” is equivalent to all other categories combined, i.e., non-spectrum and autistic disorder and pervasive developmental disorder - not otherwise specified. Figure created using Lucidchart


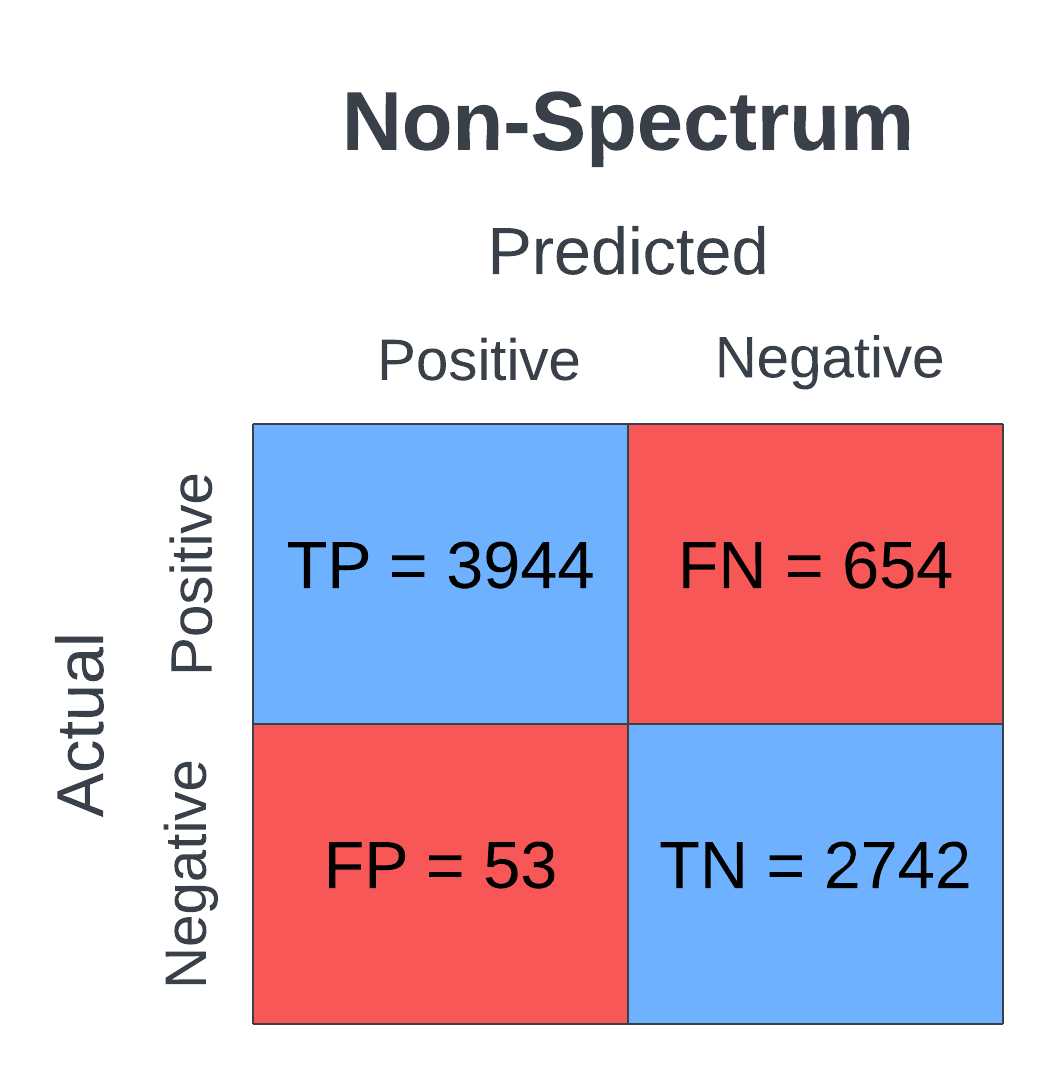


**Supplementary Fig. 13** Confusion matrix showing machine learning model output for Simons Foundation Powering Autism Research for Knowledge (SPARK) dataset for non-spectrum. “Positive” is equivalent to non-spectrum, and “Negative” is equivalent to all other categories combined, i.e., autistic disorder and Asperger’s disorder and pervasive developmental disorder - not otherwise specified. Figure created using Lucidchart


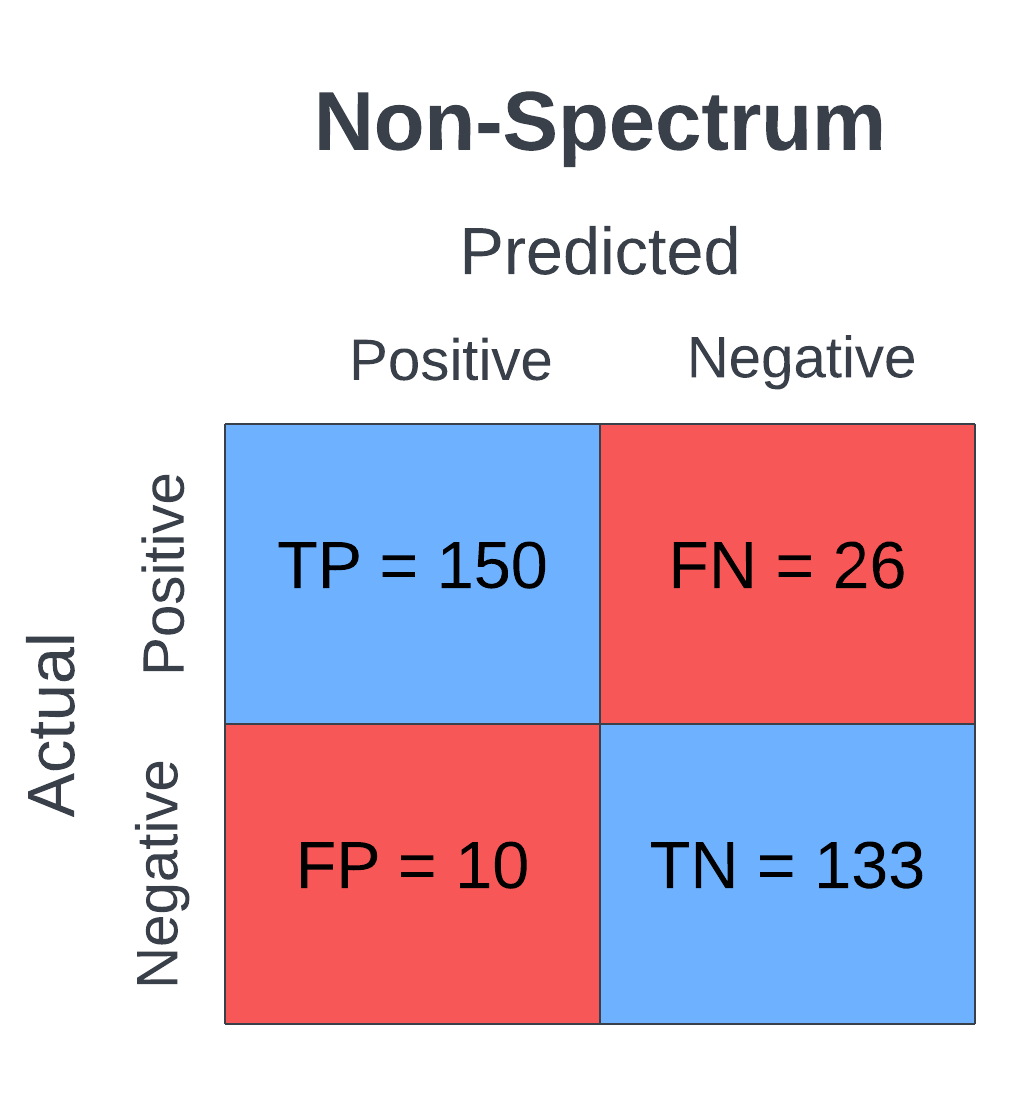


**Supplementary Fig. 14** Confusion matrix showing machine learning model output for Autism Brain Imaging Data Exchange (ABIDE) dataset for non-spectrum. “Positive” is equivalent to non-spectrum, and “Negative” is equivalent to all other categories combined, i.e., autistic disorder and Asperger’s disorder and pervasive developmental disorder - not otherwise specified. Figure created using Lucidchart
